# Pericyte FAK negatively regulates Gas6/Axl signalling to suppress tumour angiogenesis and tumour growth

**DOI:** 10.1038/s41467-020-16618-6

**Published:** 2020-06-04

**Authors:** Tanguy Lechertier, Louise E. Reynolds, Hyojin Kim, Ana Rita Pedrosa, Jesús Gómez-Escudero, José M. Muñoz-Félix, Silvia Batista, Matthew Dukinfield, Fevzi Demircioglu, Ping Pui Wong, Kylie P. Matchett, Neil C. Henderson, Gabriela D’Amico, Maddy Parsons, Catherine Harwood, Pascal Meier, Kairbaan M. Hodivala-Dilke

**Affiliations:** 1 Centre for Tumour Biology, Barts Cancer Institute – a CR-UK Centre of Excellence, Queen Mary University of London, John Vane Science Centre, Charterhouse Square, London, EC1M 6BQ UK; 2 Cell Death & Inflammation, The Breast Cancer Now Toby Robins Research Centre, Institute of Cancer Research, Fulham Road, London, SW3 6JB UK; 3 Systems Oncology Group, Champalimaud Research, Champalimaud Centre for the Unknown Av. Brasília, Doca de Pedrouços, 1400-038 Lisbon, Portugal; 4 Present Address: Guangdong Provincial Key Laboratory of Malignant Tumor Epigenetics and Gene Regulation, Medical Research Center, Sun Yat-Sen Memorial Hospital, Sun Yat-Sen University, 510120 Guangzhou, China; 5 Centre for Inflammation Research, The Queen’s Medical Research Institute, Edinburgh BioQuarter, University of Edinburgh, Edinburgh, UK; 6 MRC Human Genetics Unit, Institute of Genetics and Molecular Medicine, University of Edinburgh, Crewe Road South, Edinburgh, UK; 7 Nikon Imaging Centre@King’s, Randall Division of Cell and Molecular Biophysics, Kings College London, Room 3.22B, New Hunts House Guys Campus, London, SE1 1UL UK; 8 Centre for Cell Biology and Cutaneous Research, Blizard Institute, Barts and The London School of Medicine and Dentistry, Queen Mary University of London, London, E1 2AT UK

**Keywords:** Growth factor signalling, Cancer microenvironment

## Abstract

The overexpression of the protein tyrosine kinase, Focal adhesion kinase (FAK), in endothelial cells has implicated its requirement in angiogenesis and tumour growth, but how pericyte FAK regulates tumour angiogenesis is unknown. We show that pericyte FAK regulates tumour growth and angiogenesis in multiple mouse models of melanoma, lung carcinoma and pancreatic B-cell insulinoma and provide evidence that loss of pericyte FAK enhances Gas6-stimulated phosphorylation of the receptor tyrosine kinase, Axl with an upregulation of Cyr61, driving enhanced tumour growth. We further show that pericyte derived Cyr61 instructs tumour cells to elevate expression of the proangiogenic/protumourigenic transmembrane receptor Tissue Factor. Finally, in human melanoma we show that when 50% or more tumour blood vessels are pericyte-FAK negative, melanoma patients are stratified into those with increased tumour size, enhanced blood vessel density and metastasis. Overall our data uncover a previously unknown mechanism of tumour growth by pericytes that is controlled by pericyte FAK.

## Abstract (web summary)

Focal adhesion kinase (FAK) is required for tumour angiogenesis and growth. Here, the authors show that deletion of pericyte FAK upregulates Gas6-Axl mediated Cyr61 production, which increases endothelial cell proliferation and angiogenesis, while elevating tissue factor production to enhance tumour cell proliferation.

## Introduction

Pericytes are perivascular cells whose role, via interactions and crosstalk with endothelial cells (ECs)^1–3^, in physiological angiogenesis is well established. However, the molecular mechanisms involved in the regulation of tumour growth, by pericytes is still unknown. It is accepted that, in tumours, a mis-regulation of endothelial cell–pericyte interactions and crosstalk leads to instability of the microvasculature^4,5^ that can regulate tumour growth, progression, and metastasis^6,7^. More recently, growing evidence supports a new role for cross-talk between pericytes and tumour cells^8,9^.

The protein tyrosine kinase, focal adhesion kinase (FAK) is upregulated in many cancer types leading to the development of FAK-inhibitors that are currently in clinical trials^10^. Unfortunately, monotherapy with FAK-inhibitors shows no overall benefit and raises the question of the precise regulation of tumour growth by FAK in various cell types of the tumour. FAK is thought to regulate adhesion, proliferation, migration, and survival of ECs^11^ and the role of FAK in angiogenesis has been studied using EC *FAK* knockout animal models^12–14^. Further work has shown the requirement of EC FAK in the initiation of tumour angiogenesis^15,16^. However, the role of pericyte FAK in tumourigenesis has never been investigated.

Here we identify pericyte FAK as a negative regulator of tumour angiogenesis and tumour growth, through its control of Gas6-stimulated Axl activation. Furthermore, we have identified the relationship between pericyte FAK expression on blood vessels and tumour angiogenesis and growth in human melanoma samples. Together, these data highlight an important role for cross-talk between pericytes, ECs and tumour cells, rather than with ECs alone, in the regulation of tumour angiogenesis and growth and place pericyte FAK as an important regulator in this process.

## Results

### Pericyte FAK deficiency increases tumour growth and tumour angiogenesis

The role of pericyte FAK in tumour growth is unknown. To develop a genetic tool to assess how loss of pericyte FAK could affect tumour growth, we used Cre-lox recombination to delete FAK in *pdgfrβcre*+ cells*. Pdgfrβcre-;fak*fl/fl and *pdgfrβcre*+*;fak*fl/fl mice were born at normal Mendelian ratios, and showed no defects in weight, gender distribution or large vessel morphology in unchallenged tissue (Supplementary Fig. 1a–d). FAK expression was not affected in endothelial cells or fibroblasts from *pdgfrβcre*+*;fak*fl/fl mice but was specifically deleted in pericytes isolated from *pdgfrβcre*+*;fak*fl/fl mice, at both protein and RNA levels, with no change in *Pdgfrβ* RNA levels (Supplementary Fig. 1e, f).

**Fig. 1:**
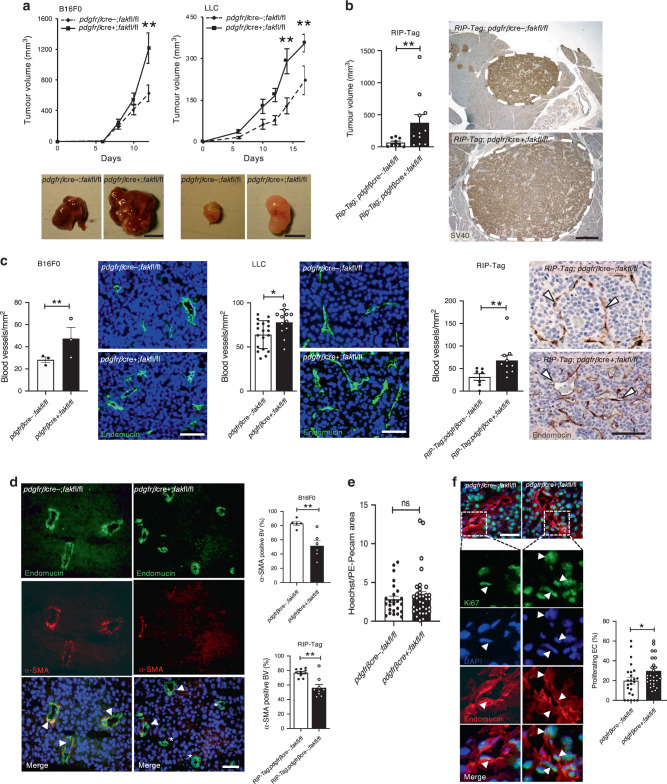
Tumour growth and angiogenesis are enhanced in *pdgfrβcre+;fakfl/fl* mice. **a** B16F0 melanoma and Lewis Lung Carcinoma (LLC) subcutaneous tumour growth was increased in *pdgfrβcre*+;*fakfl/fl* mice compared with *pdgfrβcre-;fakfl/fl* mice. Images of representative tumours. Data show mean ± s.e.m. *n* = 17 (B16F0) and 7 (LLC) *pdgfrβcre-;fakfl/fl* and 25 (B16F0) and 8 (LLC) *pdgfrβcre*+;*fakfl/fl* mice per tumour type. **p = 0.009. Scale bar, 500 μm. **b** Spontaneous pancreatic insulinoma tumour volume is increased in *RIP-Tag*;*pdgfrβcre*+;*fakfl/fl* mice compared with *RIP-Tag*;*pdgfrβcre-;fakfl/fl* mice. Chart represents mean total macroscopic tumour volume ± s.e.m. *n* = 9 *RIP-Tag*;*pdgfrβcre-;fakfl/fl* and 11 *RIP-Tag*;*pdgfrβcre*+;*fakfl/fl* mice. **p = 0.0042. Representative images of pancreatic tumours stained for SV40. Scale bar, 0.5 cm. **c** Tumour blood vessel density is increased in tumours grown in *pdgfrβcre*+;*fakfl/fl* compared with *pdgfrβcre-;fakfl/fl* mice. Charts represent mean ± s.e.m. *n* = 3 *pdgfrβcre-;fakfl/fl* and 3 *pdgfrβcre*+;*fakfl/fl* B16F0 tumours, **p = 0.009; 19 *pdgfrβcre-;fakfl/fl* and 12 *pdgfrβcre*+;*fakfl/fl* LLC tumours *p = 0.0207; 7 *RIP-Tag*;*pdgfrβcre-;fakfl/f* and 10 *RIP-Tag*;*pdgfrβcre*+;*fakfl/fl* tumours, **p = 0.0097. Representative images of endomucin stained sections. Scale bar for B16F0 and LLC tumour sections, 200 µm. Scale bar for RIP-Tag tumour sections, 300 µm. **d** Pericyte association with tumour blood vessels is reduced in *pdgfrβcre*+;*fakfl/fl* and *RIP-Tag*;*pdgfrβcre*+;*fakfl/fl* mice. Charts represent the percentage of α-SMA positive blood vessels ± s.e.m. *n* = 5 *pdgfrβcre-;fakfl/fl*, 6 *pdgfrβcre*+;*fakfl/fl*, 10 *RIP-Tag*;*pdgfrβcre-;fakfl/fl*, 9 *RIP-Tag*;*pdgfrβcre*+;*fakfl/fl* mouse tumours. **p = 0.0043 (B16F0), **p = 0.003 (RIP-Tag). **e** Blood vessel leakage was similar in tumours from *pdgfrβcre-;fakfl/fl* and *pdgfrβcre*+;*fakfl/fl* mice. Chart shows Hoechst area relative to blood vessel area ± s.e.m. *n* = 25 high power fields in tumours from 4 *pdgfrβcre-;fakfl/fl* mice and 34 fields in tumours from 4 *pdgfrβcre*+;*fakfl/fl* mice. **f** Blood vessel associated endothelial cells from *pdgfrβcre*+;*fakfl/fl* mice have increased proliferation. Lower panels show representative high power images of insert Ki67, DAPI, endomucin. Chart represents the percentage of proliferating endothelial cells per area ± s.e.m., *n* = 24 and 27 fields in 4 *pdgfrβcre-;fakfl/fl*, 5 *pdgfrβcre*+;*fakfl/fl* mouse tumours respectively. *p = 0.0457. **a**–**e** two-sided Mann–Whitney *U*-test; **c**, **d**, **f** Two-sided Student’s *t*-test; ns not significant. Source data are provided as a Source data file.

To examine the effect of pericyte FAK loss on tumour growth and angiogenesis, *pdgfrβcre*+;*fak*fl/fl and *pdgfrβcre-;fak*fl/fl mice were injected subcutaneously with either B16F0 melanoma or Lewis lung carcinoma (LLC) cells. In both models, tumours grew significantly bigger in *pdgfrβcre*+;*fak*fl/fl animals when compared with the *pdgfrβcre-;fak*fl/fl controls (Fig. 1a). To extend these effects to a spontaneous tumour model we crossed the *pdgfrβcre*+;*fak*fl/fl mice with *RIP-Tag* mice, a model of pancreatic insulinoma^17^. At 15 weeks of age, *RIP-Tag*;*pdgfrβcre*+;*fak*fl/fl mice displayed larger tumours than *RIP-Tag*; *pdgfrβcre-;fak*fl/fl controls, suggesting that the loss of pericyte FAK was also sufficient to control tumour growth in this spontaneous model of cancer (Fig. 1b). This increase in tumour growth in *pdgfrβcre*+;*fak*fl/fl mice correlated with enhanced tumour blood vessel density (Fig. 1c) that was not apparent in unchallenged skin (Supplementary Fig. 1g). Importantly, in tumour burdened *pdgfrβcre*+;*mTmG* reporter mice, the specificity of Cre expression in pericytes associated with tumour blood vessels was confirmed. In detail, blood vessels from *pdgfrβcre-;mTmG* and *pdgfrβcre*+*;mTmG* mice both displayed tomato (mT) signal in host cells, but after Cre excision GFP (mG) was only observed in *pdgfrβcre*+*;mTmG* mouse tumour pericytes (Supplementary Fig. 1h). When examining unchallenged skin *TdTom; pdgfrβcre-* mice had no apparent *TdTom* signal (data not shown). Importantly, immunofluorescence staining for Pdgfr*β* showed a weak signal in 76% of dermal vessels indicating that Pdgfr*β* is poorly expressed in pericytes of unchallenged adult mouse skin. Furthermore, *TdTomato* (*TdTom*) signal in *TdTom; pdgfrβcre*+ mice, showed *TdTom* in approximately 36% of dermal vessels. This result indicated poor *pdgfrβ*-cre activity in mural cells of dermal blood vessels (Supplementary Fig. 1i) likely explaining the lack of a blood vessel phenotype in unchallenged skin of *pdgfrβcre*+;*fak*fl/fl mice. Immunostaining for FAK confirmed in vivo pericyte-FAK loss in tumours from *pdgfrβcre*+;*fak*fl/fl mice (Supplementary Fig. 1j). These data validated that *pdgfrβ-*driven FAK deletion in vivo is restricted to tumour pericytes in the experimental settings used.

Given that FAK plays a role in cell migration and adhesion, it was not necessarily surprising that pericyte FAK deficiency reduced significantly the association of NG2-positive and α-SMA-positive (that were also shown to be Pdgfr*β*-positive) pericytes with tumour blood vessels (Fig. 1d, Supplementary Figs. 2 and  3). Despite this, there was no increase in blood vessel leakage in tumours from these mice when compared with control mice (Fig. 1e). Severe loss of pericyte association with blood vessels has been reported to affect microvessel dilation, basement membrane deposition and perfusion^18,19^. Histological examination of the tumour blood vessels showed no effect on microvessel dilation, laminin deposition, VE-cadherin localisation (Supplementary Fig. 4a–c) or perfusion between the genotypes (Supplementary Fig. 5). This suggests that decreased pericyte investment of blood vessels in *pdgfrβcre*+;*fak*fl/fl mouse tumours does not affect blood vessel leakage or perfusion. In line with the enhanced tumour angiogenesis observed in Fig. 1c, endothelial cell proliferation was increased significantly in B16F0 tumours grown in *pdgfrβcre*+;*fak*fl/fl mice when compared with control mice (Fig. 1f). Given the pro-angiogenic phenotype in the *pdgfrβcre*+;*fak*fl/fl mice, we next examined the effect on tumour immune cell infiltration, especially since F4/80+ macrophages are thought to control angiogenesis^20^. No apparent defect in CD45+ or F4/80+ immune cell infiltration was observed in subcutaneous or RIP-Tag tumours grown in *pdgfrβcre*+;*fak*fl/fl mice, suggesting that immune cell infiltration was not a major factor in tumour growth control (Supplementary Fig. 6). In short, loss of pericyte FAK, in contrast to the loss of endothelial FAK^16^, was sufficient to enhance tumour growth and angiogenesis in three models of cancer with a reduction in pericyte-microvessel association.

### Determining the molecular players in the enhanced tumour angiogenesis in *pdgfrβcre+;fakfl/fl* mice

**Fig. 2:**
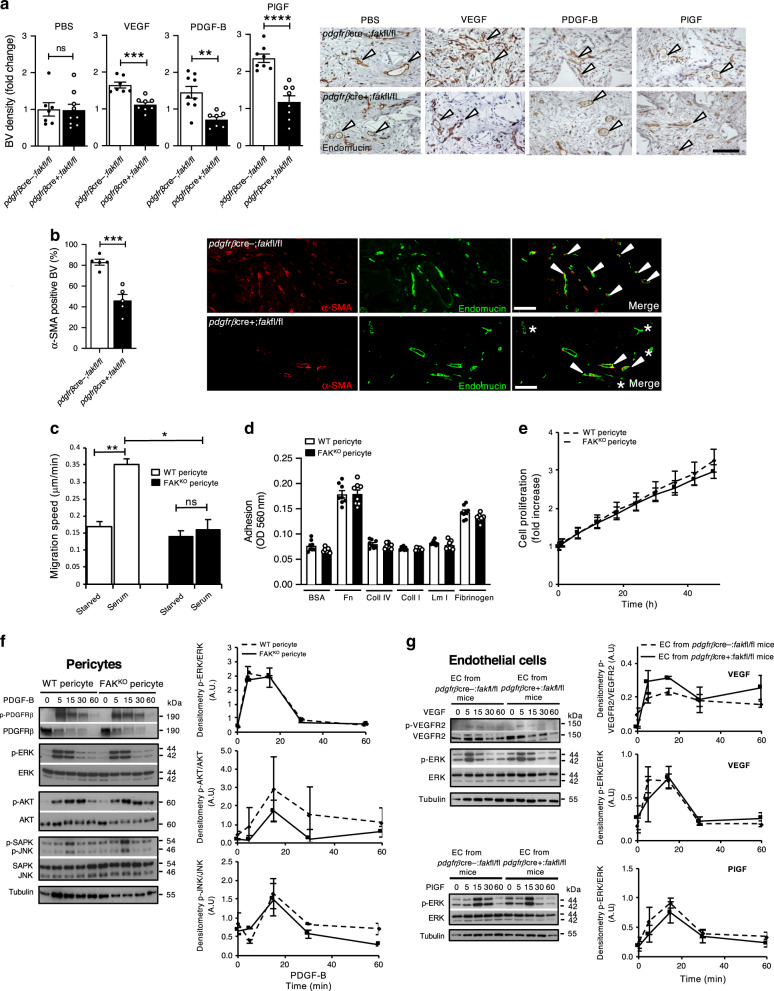
FAK deletion in pericytes inhibits angiogenesis in response to VEGF, PDGF-B and PlGF. **a** VEGF-stimulated, PDGF-B-stimulated and PlGF-stimulated angiogenesis in vivo were all reduced significantly in *pdgfrβcre*+;*fakfl/fl* mice. Charts represent quantitation of blood vessels in infiltrated areas of sponges ± s.e.m., *n* = 7 and 9 PBS and 7 and 8 VEGF treated sponges in *pdgfrβcre-;fakfl/fl* and *pdgfrβcre*+;*fakfl/fl* mice, respectively. 9 and 7 PDGF-B and 8 and 8 PlGF treated sponges in *pdgfrβcre-;fakfl/fl* and *pdgfrβcre*+;*fakfl/fl* mice, respectively; ***p = 0.0002 (VEGF), **p = 0.0019 (PDGF-B), ****p < 0.0001 (PlGF). Representative images of endomucin stained sections of sponges. Scale bar, 100 μm. **b** α-SMA-positive pericyte association with blood vessels were reduced in sponges from *pdgfrβcre*+;*fakfl/fl* mice when compared with *pdgfrβcre-;fakfl/fl* mice. Charts represent percentage of α-SMA-positive blood vessels ± s.e.m., *n* = 5 sponges each from *pdgfrβcre-;fakfl/fl* and *pdgfrβcre*+;*fakfl/fl* mice, ***p = 0.0004. Representative images of α-SMA/endomucin stained sections of sponges. Scale bar, 200 μm. **c** FAK^KO^ pericyte migration speed is decreased in response to serum. Chart represents migration speed in serum starved WT and FAK^KO^ pericytes and WT and FAK^KO^ pericytes in the presence of serum + s.e.m. **p < 0.005, *p < 0.01, *n* = 3 biological repeats in duplicate. **d** WT and FAK^KO^ pericytes showed no difference in adhesion to BSA, Fibronectin, Collagen I, Collagen IV, Laminin 1 or Fibrinogen. Chart represents the relative number of cells that attached to the matrix normalised to BSA (negative control) ± s.e.m. *n* = 8 replicates. **e** WT and FAK^KO^ pericytes prolif**e**rate at a similar rate. Line graph represents mean ± s.e.m.; *n* = 4 wells/genotype, 5 experimental repeats. **f** WT and FAK^KO^ pericytes show no differences in signalling in response to PDGF-B. Graphs represent the densitometric quantitation of p-ERK1/2/ERK1/2 ratio, p-AKT:AKT ratio and p-JNK:JNK ratio ± s.e.m.; *n* = 2 experimental repeats. **g** Endothelial cells isolated from *pdgfrβcre-;fakfl/fl* and *pdgfrβcre*+;*fakfl/fl* show no differences in signalling in response to VEGF or PlGF. Graphs represent the densitometric quantitation of p-VEGFR2/VEGFR2 with VEGF, p-ERK/ERK with VEGF and p-ERK/ERK with PlGF ratios ± s.e.m.; *n* = 3 experimental repeats. **a**–**c** Two-sided Students *t*-test, ns not significant. Source data are provided as a Source data file.

To determine which growth factors may be responsible for the enhanced tumour angiogenesis in *pdgfrβcre*+;*fak*fl/fl mice, synthetic sponges were implanted subcutaneously into *pdgfrβcre-;fak*fl/fl and *pdgfrβcre*+;*fak*fl/fl mice, and angiogenesis was stimulated with PBS (control), PDGF-B, PlGF, or VEGF. Interestingly, although no differences in PBS-stimulated angiogenesis were observed between genotypes, we did not see an increase in blood vessel density in response to PDGF-B, PlGF, and VEGF, but rather a decrease (Fig. 2a). This was despite a significant decrease in pericyte coverage of blood vessels in growth factor induced angiogenesis in *pdgfrβcre*+;*fak*fl/fl mice, as determined by α-SMA immunostaining, a known marker for pericytes (Fig. 2b). In vitro, random cell migration in response to serum was enhanced in WT pericytes but not FAK-null pericytes, suggesting that the absence of FAK abrogates serum dependent migration speed (Fig. 2c). At a cellular level, despite the loss of FAK, pericytes showed no defect in adhesion to various matrices including fibronectin, collagen I, collagen IV, laminin 1 or fibrinogen (Fig. 2d) and also showed no defect in cellular proliferation (Fig. 2e) or expression of other focal contact proteins including vinculin, talin, paxillin and phospho-paxillin (Supplementary Fig. 7a). Since PDGF-B stimulation enhances pericyte migration and proliferation through activation of PDGFR*β*, we examined PDGF-B stimulated signalling in WT and FAK^KO^ pericytes. Levels of PDGFR*β*, p-PDGFR*β*, ERK1/2 and p-ERK1/2, AKT and p-AKT, JNK/SAPK and p-JNK/SAPK were similar between the two genotypes (Fig. 2f). Although FAK deletion was not apparent in endothelial cells isolated from *pdgfrβcre*+;*fak*fl/fl mice, since our in vivo results showed that endothelial cell proliferation was enhanced in tumours grown in these mice, we examined VEGF-receptor 2 (VEGFR2) levels and downstream ERK1/2 phosphorylation in endothelial cells isolated from *pdgfrβcre-;fakfl/fl* and *pdgfrβcre*+;*fakfl/fl* mice. There were no differences in VEGF-stimulated p-VEGFR2:VEGFR2 or p-ERK1/2:ERK1/2 levels between the endothelial cells from *pdgfrβcre-;fak*fl/fl or *pdgfrβcre*+;*fak*fl/fl mice or between endothelial cells stimulated with PlGF, indicating that the enhanced endothelial cell proliferation in tumours grown in *pdgfrβcre*+;*fak*fl/fl mice was due to secondary effects of pericyte FAK-deficiency (Fig. 2g). These results show that deletion of pericyte FAK does not affect PDGF-B mediated-signalling and that ECs from *pdgfrβcre-;fak*fl/fl and *pdgfrβcre*+;*fak*fl/fl mice are not intrinsically affected either after VEGF, or PlGF, stimulation. Together, these data suggest that other pro-angiogenic factors apart from VEGF, PDGF-B and PlGF are involved in the enhanced tumourigenesis in *pdgfrβcre*+;*fak*fl/fl mice.

### Exogenous Gas6 enhances angiogenic responses via pericyte Axl in FAK^KO^ pericytes

**Fig. 3:**
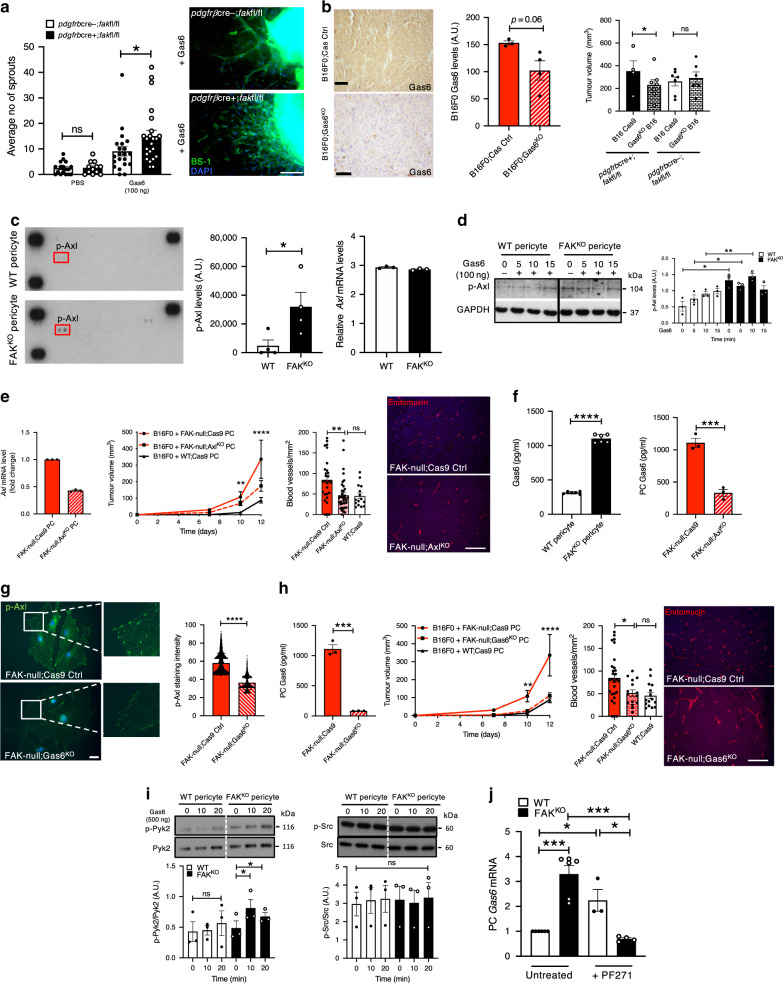
Exogenous Gas6 enhances angiogenic responses in FAK^KO^ pericytes. **a** Aortic vessel sprouting quantitation. Graphs mean ± s.e.m.; *n* = 17 and 12 PBS treated *pdgfrβcre-;fakfl/fl* and *pdgfrβcre*+;*fakfl/fl* rings respectively, and 21 and 23 Gas6 treated *pdgfrβcre-;fakfl/fl* and *pdgfrβcre*+;*fakfl/fl* aortic rings respectively; *p = 0.04. Images of Gas6 stimulated microvessels. Scale bar, 100 μm. **b** Gas6 expression in B16F0 tumours. Chart, mean ± s.e.m; *p* = 0.06, *n* = 3 and 4 sections for CasCtrl and Gas^KO^ respectively. Scale bar, 50 μm. Tumour size measurements. Chart, mean ± s.e.m., *p = 0.011; *n* = 11 *pdgfrβcre*+;*fakfl/fl* and 13 *pdgfrβcre-;fakfl/fl* mice. **c** Cytokine array, p-Axl quantitation. Chart, mean ± s.e.m., *p = 0.0467; *n* = 4 technical repeats. mRNA *Axl* levels unchanged. Chart, *Axl* mRNA, *n* = 3 biological repeats. **d** Western blot, p-Axl in Gas6-stimulated pericytes. Densitometry chart, mean ± s.e.m. *n* = 3 experimental repeats/genotype. *p = 0.0137, **p = 0.0069. **e** *Axl* mRNA-depletion. *n* = 3 experimental repeats. Chart, *Axl* mRNA fold change. Tumour growth (mm^3^): Graph, mean ± s.e.m., **p = 0.0046, ****p < 0.0001; FAK-null;Axl^KO^ PC, *n* = 8 mice; Cas6 Ctrl; *n* = 5  mice, WT Ctrl; *n* = 10 mice. Tumour blood vessel density: Chart, mean ± s.e.m., **p = 0.0017. *n* = 28, 32 and 14 fields of view for FAK-null;Axl^KO^ PC, *n* = 4 mice; Cas6 Ctrl; *n* = 4 mice, WT Ctrl; *n* = 3 mice respectively. Endomucin staining of tumour sections. Scale bar, 50 μm. **f** Gas6 expression in pericytes. Charts, mean ± s.e.m., Left chart, *n* = 6 biological repeats/genotype ****p < 0.0001, ***p = 0.0009; Right chart *n* = 3 biological repeats/genotype. **g** Pericytes p-Axl immunostaining. Chart, p-Axl median staining intensity. ****p < 0.0001, two-sided Mann–Whitney *U*-test, *n* = 4 stained slides/genotype. Scale bar, 25 μm. (**h**) Pericyte Gas6. Chart, mean ± s.e.m., ***p = 0.0001; *n* = 3 biological repeats/genotype. Tumour growth: Line graph, mean ± s.e.m., **p = 0.0046, ****p < 0.0001. FAK-null;Gas6^KO^: *n* = 14 mice, FAK-null;Cas9: *n* = 5 mice, WT;Cas9; *n* = 10 mice. Tumour blood vessel density; Chart, mean ± s.e.m., *p = 0.0179. *n* = 28, 15 and 15 fields of view for FAK-null;Gas6^KO^: *n* = 4 mice, FAK-null;Cas9: *n* = 3 mice, WT;Cas9; *n* = 3 mice respectively. Tumour endomucin immunostaining. Scale bar, 50 μm. **i** Western blots; Pericyte p-Pyk2/Pyk2 and p-Src/Src. Densitometric ratios, mean ± s.e.m., *n* = 3 experimental repeats/genotype. *p = 0.047 (FAK^KO^ 0 vs. FAK^KO^ 10), *p = 0.045 (FAK^KO^ 0 vs. FAK^KO^20). **j** *Gas6* mRNA quantitation. Chart, mean ± s.e.m, *n* = 3–4 biological repeats for PF271 treated cells and 6 for untreated cells. ****p < 0.0006 (WT vs. FAK^KO^), *p = 0.0127 (WT vs. WT + PF271), *p = 0.0216 (WT + PF271 vs. FAK^KO^ + PF271), ***p = 0.0008 (FAK^KO^ vs. FAK^KO^ + PF271). **a**–**f**, **h**–**j** two-sided Students *t*-test; **e**, **h** one-way ANOVA; ns not significant. Source data are provided as a Source data file.

In addition to VEGF, PDGF-B and PlGF, Gas6 is also highly upregulated in cancer cells. Gas6 can be expressed at nanomolar levels, and is thought to have proangiogenic functions, but the regulation of Gas6-mediated angiogenesis remains relatively obscure^21,22^. In ex vivo assays, stimulation with recombinant Gas6 (100 ng), but not PBS, enhanced sprouting angiogenesis in aortic rings from *pdgfrβcre*+;*fak*fl/fl mice compared with *pdgfrβcre-;fak*fl/fl mice (Fig. 3a). These data suggested that exogenous Gas6 stimulated responses are elevated by the loss of pericyte FAK. Moreover, CRIPSR-Cas 9 depletion of Gas6 in B16 cells reduced the enhanced tumour growth in *pdgfrβcre*+;*fak*fl/fl mice, but had no significant effect in *pdgfrβcre-;fak*fl/fl mice, indicating that tumour cell derived Gas6 is involved in the enhanced tumour responses in *pdgfrβcre*+;*fak*fl/fl mice (Fig. 3b).

In line with these results, *Protein profiler* array analysis identified phospho-Axl (p-Axl), a member of the TAM (Tyro3, Axl and Mer) family of receptor tyrosine kinases and the major receptor for Gas6, as the most significantly upregulated phospho-receptor tyrosine kinase in FAK^KO^ pericytes when compared with WT pericytes, despite no change in transcript levels of Axl (Fig. 3c). These results suggested that constitutively elevated p-Axl in FAK^KO^ pericytes could be responsible for priming these cells to be hyper-responsive to exogenous Gas6. Indeed, western blot analysis confirmed that FAK^KO^ pericytes had significantly increased p-Axl levels which were maintained after Gas6 (100 ng/ml) stimulation up to 10 min after stimulation (Fig. 3d). The binding affinity of Axl to Gas6 has been reported to be significantly higher than for Tyro3^23^, and although p-Tyro3 levels were also increased in FAK^KO ^pericytes (Supplementary Fig. 7b) we focused on pericyte Axl as the major Gas6 receptor in the enhanced Gas6-stimulated angiogenic responses controlled by pericyte FAK.

To test the functional requirement of pericyte Axl in the enhanced tumour growth and angiogenesis regulated by FAK, Axl was depleted by CRISPR-Cas9 gene editing in FAK^KO ^pericytes (FAK-null;Axl^KO^ pericytes). In vivo, the effect of targeting *Axl* in FAK^KO^ pericytes was tested by co-injecting wildtype C57bl6 mice with B16F0 tumour cells and either FAK-null;Axl^KO^, FAK-null;Cas9 or WT;Cas9 control pericytes. Depletion of Axl in FAK^KO^ pericytes reduced the enhanced tumour growth and angiogenesis observed in mice co-injected with FAK-null;Cas9 pericytes (Fig. 3e) suggesting that the elevated p-Axl in FAK^KO^ pericytes is likely involved in the enhanced tumour growth in *pdgfrβcre*+;*fakfl/fl* mice.

A previously published study indicated that Axl cross-regulates Gas6 expression in the same cell type and that this co-regulation of endogenous Axl and Gas6 is important in subsequent downstream signalling^24^. Supporting this and correlating with the enhanced p-Axl expression levels observed in FAK^KO^ pericytes, Gas6 expression was increased significantly (high picomolar levels) in FAK^KO^ pericytes compared with WT pericyte controls. Additionally, pericyte Gas6 expression levels were reduced by Axl-depletion in FAK^KO ^pericytes (Fig. 3f). Conversely, Gas6-depletion in FAK^KO^ pericytes also resulted in a significant reduction in p-Axl in FAK-null;Gas6^KO^ pericytes, measured by p-Axl staining intensity (Fig. 3g). Together these data suggest that Axl and Gas6 cross regulate their expression levels in pericytes. In vivo, the loss of Gas6 in FAK^KO^ pericytes also reduced tumour size and angiogenesis in mice co-injected with FAK-null;Gas6^KO^ and B16F0 tumour cells compared with mice co-injected with FAK-null;Cas9 pericytes and B16 tumour cells (Fig. 3h). Thus, together these data suggest that in the absence of exogenous Gas6, pericyte-derived Gas6 is not sufficient to affect angiogenesis and this is illustrated by the ex vivo assays in Fig. 3a. However, in vivo, tumour derived Gas 6 responses are enhanced, due to elevated Axl/Gas6-signallling in FAK^KO^ pericytes, and this response results in the enhanced tumour growth in *pdgfrβcre*+;*fakfl/fl* mice.

Whilst loss of FAK has been shown to correlate with compensation by its family member Pyk2^25^, cross-regulation of Gas6/Axl with Pyk2 and FAK, but not Src, have been reported previously in cancer cells^26^. Corroborating these results, Western blot analysis showed that p-Pyk2 was elevated in FAK^KO^ pericytes with no apparent effect on p-Src (Fig. 3i). Using PF-562,271, a FAK/Pyk2 dual kinase inhibitor, we showed that in WT pericytes inhibition of FAK/Pyk2 led to increased *Gas6* levels. Inhibition of Pyk2 in FAK^KO^ pericytes, however, caused a significant reduction in pericyte *Gas6* transcript levels, suggesting a putative mechanism by which loss of pericyte FAK is compensated by elevated p-Pyk2 in the regulation of endogenous Gas6/Axl (Fig. 3j). These results show (i) that FAK regulates Gas6 levels and (ii) kinase inhibition can partially mimic the effect of genetic ablation of FAK.

### Exogenous Gas6 stimulates angiogenesis by elevating Cyr61 in FAK^KO^ pericytes

**Fig. 4:**
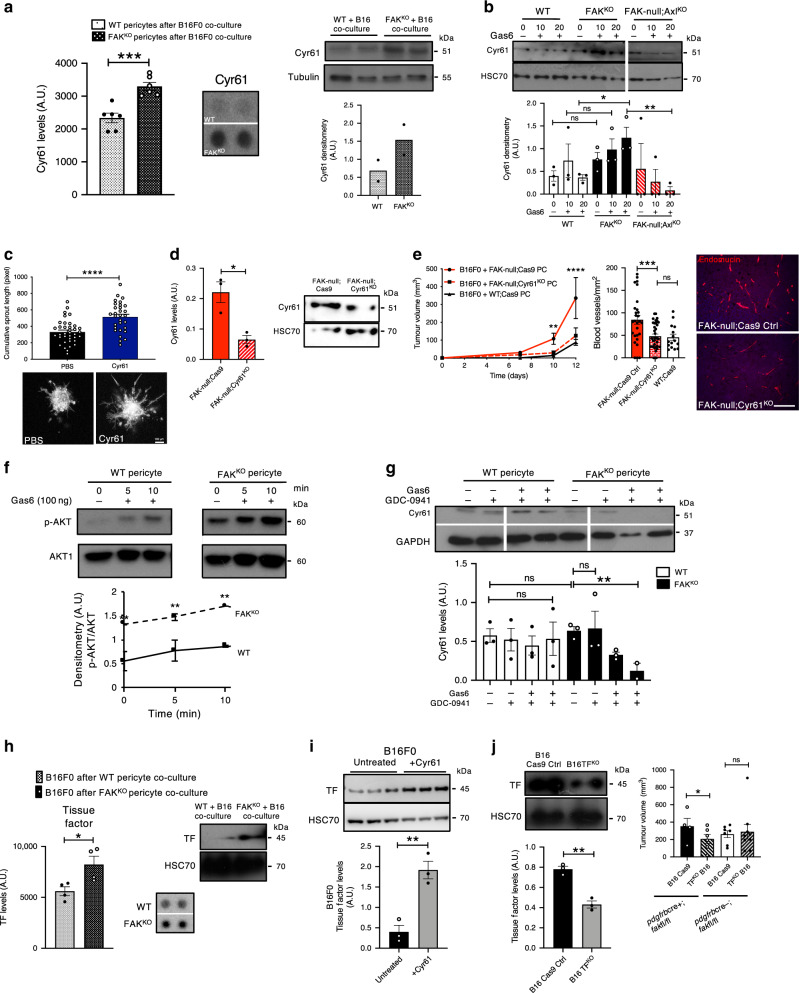
Pericyte FAK deficiency stimulates increased levels of Tissue Factor expression in B16F0 cells. **a** Cytokine array. Cyr61 quantitation in FAK^KO^ pericytes. Chart represents mean ± s.e.m. Representative images of Cyr61dots on array. *n* = 3 biological repeats in duplicate. ***p = 0.0005. This result was confirmed by western blotting of Cyr61 with the same lysates. Chart represents mean Cyr61 densitometry, *n* = 2 experimental repeats. **b** Western blot. Cyr61 expression is increased in FAK^KO^ pericytes. Depletion of Axl (FAK-null;Axl^KO^) abrogated Cyr61 expression. Chart represents mean ± s.e.m., *n* = 3 biological repeats. *p = 0.0213, **p = 0.009. **c** Recombinant Cyr61 stimulated endothelial cell spheroid sprouting. Chart represents mean ± s.e.m., ****p < 0.0001. Scale bar, 100 μm. *n* = 37 PBS treated spheroids and 30 Cyr61 treated spheroids. **d** Western blot. Cyr61-depletion in FAK-null;Cyr61^KO^ pericytes. Chart represents mean Cyr61 levels ± s.e.m., *p = 0.0133; *n* = 3 experimental repeats. **e** Tumour growth. Line graph represents mean ± s.e.m., **p = 0.0046, ****p < 0.0001. FAK-null;Cyr61^KO^: *n* = 7 mice, FAK-null;Cas9: *n* =  5 mice, WT;Cas9: *n* = 10 mice. Tumour blood vessel density. Chart represents mean blood vessel density/mm^2^ ± s.e.m., ***p = 0.0006. *n* = 28, 30 and 14 fields of view from 4 FAK-null;Cas9 tumours; *n* = 4 FAK-null;Cyr61^KO^ tumours, and *n* = 3 WTCas9 tumour sections respectively. Scale bar, 50 μm. Immunostaining for endomucin. **f** Western blot. p-AKT expression in WT and FAK^KO^ pericytes. Line graph shows mean ± s.e.m., **p = 0.0043; *n* = 3 experimental repeats. **g** Western blot. PI3-kinase inhibitor (GDC-0941) reduced expression of Cyr61 after stimulation with Gas6 in FAK^KO^ pericytes (10 and 20 min). Chart represent mean ± s.e.m Cyr61 levels. **p = 0.0047; *n* = 3 biological repeats. **h** Cytokine array. Tissue factor (TF) quantification. Representative images of dot blots. Chart represents mean ± s.e.m.TF levels, *n* = 4 biological repeats. *p = 0.0295. Western blot analysis confirmed increased TF production in B16F0 melanoma cells co-cultured with FAK^KO^ pericytes. **i** Western blotting. B16F0 cells had increased expression of TF in response to exogenous Cyr61 (10 μg/ml). Chart represents mean ± s.e.m. *n* = 3 experimental repeats. **p = 0.0048. **j** Western blot. TF expression is reduced after TF depletion in B16F0 cells. Chart represents mean ± s.e.m., *n* = 3 lysates. **p = 0.0016. Tumour volume measurements. Chart represents mean ± s.e.m., *p = 0.0453; *n* = 10 *pdgfrβcre*+;*fakfl/fl* and 16 *pdgfrβcre-;fakfl/fl* mice**. a**–**d**, **f**, **g**–**j** two-sided Students *t* test. **e**, **j** one-way ANOVA; ns not significant. Source data are provided as a Source Data file.

The molecular regulation induced by exogenous Gas6-stimulation was further explored in order to identify the precise factor(s) that control the enhanced angiogenesis when pericyte FAK is deleted. In unstimulated conditions no apparent changes in angiogenesis modulators, including *Vegf-receptor 2* were observed in FAK^KO^ vs. WT pericytes (Supplementary Fig. 8a, b) supporting the idea that exogenous stimulation was required for the observed upregulation of angiogenesis. Of note, endothelial cells from *pdgfrβcre*+;*fak*fl/fl and *pdgfrβcre-;fak*fl/fl mice showed no change in p-Axl levels and had no detectable levels of Tyro3 also reinforcing the hypothesis that pericyte FAK loss did not constitutively affect endothelial cells in the absence of exogenous stimulation (Supplementary Fig. 8c). However, stimulation of pericytes by co-culture with B16F0 cells upregulated the expression of several pro-angiogenic proteins, including Cyr61, in FAK^KO^ pericytes compared with WT pericytes (Fig. 4a and Supplementary Fig. 9a). Indeed, exogenous Gas6 stimulation (100 nM) enhanced Cyr61 expression in FAK^KO^ but not WT pericytes and this increase in Cyr61 expression was abolished by deletion of Axl in FAK^KO^ pericytes (Fig. 4b). Together these results indicated that the increased levels of Gas6 expression in FAK^KO^ pericytes are not sufficient to stimulate Cyr61 expression and that exogenous stimulation is required.

Cyr61 is known to be involved in regulating cell proliferation through interaction with integrins expressed on endothelial cells and tumour cells^27–30^. Endothelial spheroid sprouting assays corroborated that stimulation with recombinant Cyr61 is proangiogenic (Fig. 4c). To test the requirement for pericyte Cyr61 in tumour growth FAK-null;Cyr61^KO^ pericytes were generated by CRIPSR-Cas9 gene editing (Fig. 4d). Co-injection of B16F0 cells with FAK-null;Cyr61^KO^ pericytes into wild type mice reduced significantly the enhanced tumour growth and angiogenesis observed after co-injection of B16F0 cells with FAK-null;Cas9 control pericytes (Fig. 4e). Cyr61 deletion in FAK^KO ^pericytes also had no effect on endogenous, pericyte Gas6 expression levels placing pericyte Gas6 up-stream of Cyr61 (Supplementary Fig. 9b). These data established that the elevated expression of Cyr61, downstream of pericyte Gas6/Axl, in FAK^KO^ pericytes is involved in the regulation of angiogenesis and tumour growth in vivo.

Signalling downstream of Axl is known to be mediated via multiple signalling pathways including PI3K/Akt/mTOR, MEK/ERK and NF-KB pathways^31,32^. Our data show that whilst exogenous Gas6-stimulation enhanced both p-Akt and p-p65 NFkB expression (Fig. 4f and Supplementary Fig. 10a, respectively), depletion of pericyte Gas6 or Axl in FAK^KO ^pericytes reduced p-AKT/AKT levels dramatically (Supplementary Fig. 10b) demonstrating that the enhanced AKT activation in exogenous Gas6-stimulated FAK^KO^ pericytes requires pericyte Gas6 and Axl.

Despite this observation, co-injection of FAK-null;AKT^KO^ pericytes (Supplementary Fig. 10c) with B16F0 tumour cells did not affect the enhanced tumour growth or angiogenesis compared with co-injection of FAK-null;Cas9 pericytes with B16F0 tumour cells (Supplementary Fig. 10d, e). However, previously published studies have indicated that genetic deletion of AKT can induce compensation by multiple alternative pathways overcoming the loss of AKT and our data suggest that this may be a limitation of this approach in understanding the relevance of pericyte AKT in vivo. As an alternative, we inhibited AKT in WT and FAK^KO^ pericytes using the PI3-kinase inhibitor GDC-0941 and stimulated or not with Gas6. AKT inhibition in the absence of Gas6 did not affect Cyr61 levels compared with basal conditions, implying that despite the increased in basal p-AKT observed in FAK^KO^ pericytes, it is not enough to stimulate Cyr61. However, in FAK^KO^ pericytes, the addition of exogenous Gas6 and AKT inhibition did significantly reduce Cyr61, which was not observed in WT pericytes, implying a requirement for exogenous Gas6 in the regulation of Cyr61through AKT (Fig. 4g).

In addition to the effect of Cyr61 on endothelial cells in regulating angiogenic responses, previous reports have indicated that Cyr61 directly affects tumour cells promoting cancer cell invasion and overexpression of Cyr61 enhances tumour growth^33–35^. Thus, we next examined the mechanism(s) by which pericyte signals could affect tumour cells directly. Co-culture of FAK^KO^ pericytes with B16F0 cells enhanced expression of B16F0 coagulation factor III/tissue factor (TF), and, more modestly, Serpin E1 compared with B16F0 cells after co-culture with WT pericytes (Fig. 4h and Supplementary Fig. 11). TF is a pleotropic factor that is involved in accelerating tumour growth and further enhancing angiogenesis^36^. Serpin E1 is a secreted protein and regulator of urokinase and tissue-type plasminogen activators. It is involved in the promotion of invasion by degradation of extracellular matrix and activation of matrix metalloproteinase^37^. Increased tissue expression of Serpin E1 in stomach and lung cancer correlates with poor prognosis^38^. B16F0 tumour cell stimulation with Cyr61 enhanced tumour cell TF expression, suggesting that the elevated Cyr61 produced by FAK^KO ^pericytes may be sufficient to directly affect tumour cell TF expression (Fig. 4i). To determine the contribution of B16F0 TF to tumour growth, TF was depleted in B16 cells and these cells (B16;TF^KO^) injected into *pdgfrβcre*+;*fak*fl/fl and *pdgfrβcre-;fak*fl/fl mice. Depletion of TF in B16F0 cells was sufficient to reduce the enhanced tumour growth in *pdgfrβcre*+;*fak*fl/fl and had no effect on tumour growth in *pdgfrβcre-;fak*fl/fl mice (Fig. 4j). Together these data demonstrate that Cyr61 stimulation from FAK-null pericytes is sufficient to enhance B16F0 TF expression and that deletion of B16F0 TF can rescue the enhanced tumour growth in *pdgfrβcre*+;*fak*fl/fl mice.

### Reduced expression of pericyte FAK is associated with human melanoma progression

**Fig. 5:**
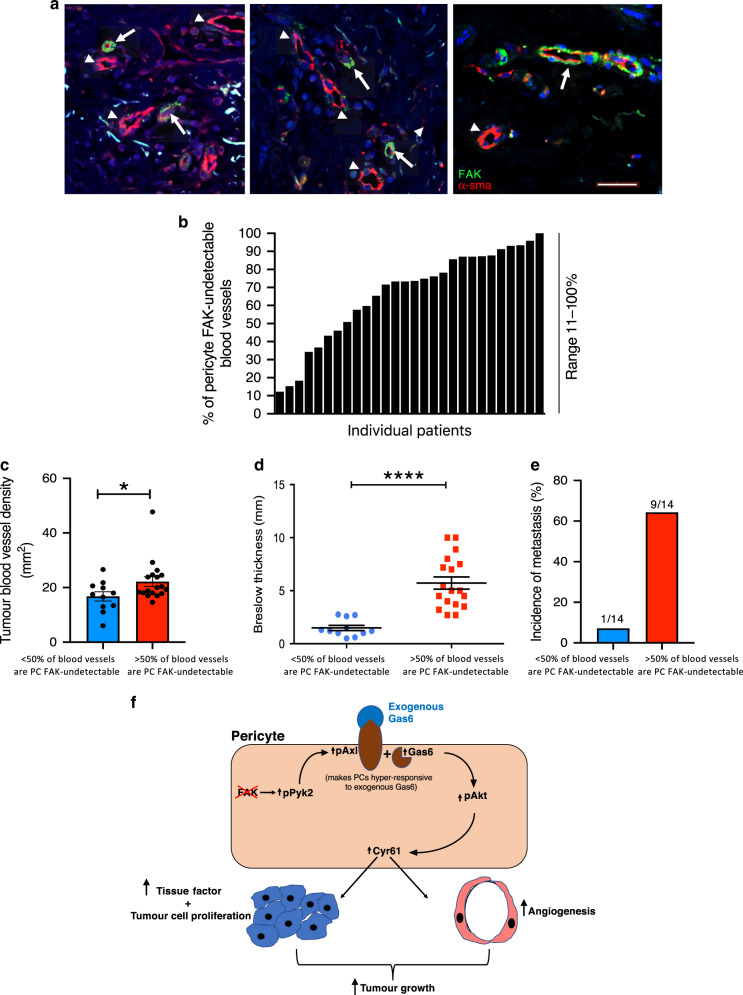
High numbers of pericyte FAK-negative blood vessels are associated with enhanced tumour size and progression in human melanoma. **a** Representative images of human melanoma showing both pericyte FAK-positive (arrows) and FAK–negative (arrowheads) blood vessels. Scale bar, 40 μm. **b** Chart represents the range of percentages of blood vessels that are pericyte-FAK negative for individual patients. *n* = 28 patient samples. **c** Tumour blood vessel density is increased in human melanoma where more than 50% of tumour blood vessels are pericyte FAK-negative. Chart represents mean ± s.e.m., *n* = 28 clinical samples, *p = 0.048. **d** Breslow thickness, an indicator of tumour burden is significantly increased in human melanoma where more than 50 % of tumour blood vessels are mural FAK negative. Chart represents mean Breslow thickness (mm) ± s.e.m., ****p < 0.0001; *n* = 28 clinical samples. **e** The incidence of metastasis is significantly increased when more than 50% of tumour blood vessels are PC FAK-negative. Chart represents incidence of metastasis (%); *n* = 14 clinical samples. **f** Schematic diagram of proposed molecular mechanism of FAK^KO^ pericyte regulation of tumour growth. Loss of pericyte FAK elevates pPyk2-Gas6-Axl-AKT signalling and subsequent Cyr61 expression that enhances angiogenesis. In parallel, Cyr61 is sufficient to enhance TF expression in tumour cells, thus enhancing tumour growth. **c**, **d** Two-sided Students *t*-test. Source data are provided as a Source data file.

Finally, we established that pericyte FAK loss was relevant to human melanoma progression. Primary human cutaneous melanoma sections were double immunostained for the pericytes and FAK to determine levels of pericyte FAK, and the percentage of blood vessels in which pericyte FAK was not detectable was established. Results revealed that pericyte FAK was detected only on a sub-set of blood vessels (Fig. 5a). The percentage of pericyte FAK undetectable blood vessels ranged between 11 and 100% across individual patient samples (Fig. 5b). Similar to our mouse data, we showed that a high proportion of pericyte FAK-undetectable blood vessels (i.e., when more than 50% of the vessels are pericyte FAK-undetectable) stratifies patients into those with increased blood vessel density (Fig. 5c), enhanced tumour burden (identified by increasing Breslow thickness) (Fig. 5d) and increased metastasis (Fig. 5e). Together these data indicate that a high percentage of blood vessels where pericyte FAK is undetectable is associated with enhanced tumour size and progression in human melanoma, and implies that pericyte FAK expression is protective in human cancer.

In summary, loss of pericyte FAK is compensated by an increase in pericyte p-Pyk2 which enhances pericyte p-Axl and concomitantly pericyte Gas6 expression. This primes FAK^KO^ pericytes to be more responsive to exogenous Gas6, derived from tumour cells, driving sustained activation of pericyte AKT and enhanced Cyr61 expression. This pericyte-derived Cyr61 is sufficient to enhance tumour growth and angiogenesis whilst also promoting TF expression perpetuating the accelerated tumour growth and angiogenesis in vivo (Fig. 5f).

## Discussion

In contrast to previous studies in which FAK was deleted in ECs and resulted in a reduction in tumour growth in multiple mouse models of cancer^15,16,39,40^ our results demonstrate that loss of pericyte FAK enhances tumour growth and tumour angiogenesis. Previously, pericytes have been shown to play critical roles in vascular development, the control of vessel contraction, blood flow and haemostasis^1,41^ but their roles beyond this are poorly defined. Indeed, although pericyte FAK loss reduces pericyte-blood-vessel association this is not sufficient to explain the enhanced tumour angiogenesis and growth in *pdgfrβcre*+;*fakfl/fl* mice.

Gas6 and Axl, known to co-regulate their expression, are expressed in pericytes^24^ and upregulated in many human cancers^21,22,42,43^, but the role of Axl signalling during angiogenesis in pericytes was unknown. Overall our study identifies that pericyte FAK expression protects against enhanced tumour growth via the regulation of pericyte Gas6-Axl-AKT-signalling pathway and subsequent control of Cyr61. Cyr61 is largely secreted by pericytes^44,45^ and is implicated in tumour growth and angiogenesis^29,33,39,46^ through stimulating EC proliferation/migration^28^. Thus, the overexpression of pericyte Cyr61 in FAK^KO^ pericytes is likely to be part of the mechanism by which loss of pericyte FAK can enhance tumour angiogenesis. Additionally, since we showed that Cyr61 can upregulate TF expression in tumour cells, and TF is known to drive tumour growth^47^, our data reveal a parallel mechanism by which pericyte regulation of Cyr61 and subsequent tumour cell TF drives accelerated tumour growth in the absence of pericyte FAK in vivo. This establishes a new molecular mechanism whereby pericyte-tumour cell cross-talk in addition to pericyte-endothelial cell cross-talk could control tumour angiogenesis and tumour growth.

Interestingly, FAK kinase inhibition, using PF-271, also partially mimics pericyte FAK deletion but the clinical impact of this result if currently unknown.

Our data emphasise that FAK has pleotropic roles in the regulation of cancer and angiogenesis in both preclinical models and clinical samples.

## Methods

### Mice

Floxed *FAK* mice (FAKfl/fl) with two LoxP sites flanking the exon that encodes FAK amino acids 413–444 were crossed with mice that express Cre recombinase under the control of the platelet derived growth factor receptor ß (*pdgfrß*) promoter to generate *pdgfrβcre*+;*fakfl/fl* and *pdgfrβcre−;fakfl/fl* mice. *pdgfrβcr*+*/−;fakfl/fl* mice were crossed with *RIP-Tag2* mice generating *RIP-Tag;pdgfrß cre-;FAKfl/fl* and *RIP-Tag;pdgfrß cre*+;*FAKfl/fl* mice. For animals bred in-house, health screens (quarterly) were conducted in accordance with FELASA guidelines for health monitoring of rodent colonies, to confirm their free status of known pathogens in accordance with FELASA screens. No clinical signs were detected. Animals were housed in groups of 4–6 mice per individually ventilated cage in a 12 h light dark cycle (06:30–18:30 light; 18:30–06:30 dark), with controlled temperature (21 ± 1 °C) and relative humidity (40–60%). The cages contained 1–1.5 cm layer of animal bedding, and with environmental enrichment including cardboard box-tunnel and crinkled paper nesting material. Animals had access to food and water ad libitum. All mice were maintained on a mixed C57/Bl6J/129 background. Both male and female mice were used, between 8 and 10 weeks of age, for all experiments.

### In vivo tumour growth

Age- and sex-matched *pdgfrcreß+;fakfl/fl* and wild-type littermate controls (*pdgfrcreß-;fakfl/fl*) were given a single subcutaneous injection into the flank of either 1 × 10^6^ B16F0 melanoma cells or 0.5 × 10^6^ Lewis lung carcinoma cells (ATCC). Tumour dimensions were measured over time and tumour growth was determined using the formula: length × width^2^ × 0.52. When tumours reached the maximum legal size allowed, mice were killed, tumour sizes measured and tumour samples were either snap-frozen or fixed in 4% paraformaldehyde (PFA) for histological analysis. *RIP-Tag;pdgfrßcre-;fakfl/fl* and *RIP-Tag;pdgfrßcre*+;*fakfl/fl* mice were killed and tumours harvested at 15 weeks. The total macroscopic tumour volume was measured. Tumours were harvested and fixed in 4% PFA for histological analysis. Blood vessel density was assessed by counting the total number of endomucin (V.7C7, sc-65495, Santa Cruz, 1:100) stained blood vessels per mm^2^ across entire midline tumour sections of size- and age-matched B16F0, LLC and Rip-Tag pancreatic tumours. To examine tumour blood vessel leakage, an intravenous injection of Hoechst (100 µl at 4 mg/ml) (14533, Sigma) was given 1 min before mice were killed. ImageJ^TM^software was used to quantify the Hoechst positive area per optical field and leakage was normalised to perfused blood vessel density stained with PE-PECAM (MEC13.3, 102507, Biolegend, 1:100). For blood vessel perfusion and blood vessel diameter measurements in tumours see Supplementary Methods.

### Design and construction of CRISPR/Cas9-EGFP plasmids

sgRNAs were designed using the CRISPOR algorithm (http://crispor.tefor.net). sgRNA sequences targeting Akt1 (Gene ID: 11651; CATTGAGCGCACCTTCCATG), Gas6 (Gene ID: 14456;B16- CCGCGCCTACCAAGTCTTCG. PC—GTCGTTCTCGAACACCTCTC), Axl (Gene ID: 26362; TCGAAGCCACACCACCTCAG), Ccn1/Cyr61 (Gene ID: 16007; CCGACTCCCGGGGCGCACTT) and F3/Tissue Factor (TF) (Gene ID: 14006; ACAGTGTAGGTATAGTTGGT) were cloned into the Lenti-CRISPR–EGFP plasmid (Addgene ID: 75159) using BsmBI enzyme site.

### Transient Neon electroporation

5.0 × 10^5^ cells were centrifuged at 300×*g* for 5 min and re-suspended in 100 μl of R1 buffer (Invitrogen). CRISPR/Cas9-EGFP plasmid DNA (10 μg) were added into the cells and loaded into a 100 μl Neon electroporation tip (Invitrogen). Electroporations were performed using 1350 mV for 20 ms with 2 pulse programme for pericytes and 1300 mV for 20 ms with 2 pulse programme for B16F0 on the Neon Electroporator (Invitrogen). After electroporation, cells were rescued in pre-warmed supplemented media and plated on 0.1% gelatin with collagen and fibronectin-coated plates for 2 days.

### Enriched CRISPR/Cas9-KO cells by flow cytometry

Cells were washed with phosphate-buffered saline (PBS) and harvested with 200 μl of FACS buffer (1% BSA and 0.5 mM EDTA in PBS) 48h-post transfection. A 488-nm diode laser was used for the detection of EGFP. In each sample, viable singlet cells were gated via forward-scatter (FSC) laser and side-scatter (SSC) and EGFP positive cells, regardless of expression levels, were sorted using a FACS AriaIII flow cytometer (BD Biosciences) at the Chelsea flow cytometry and light microscopy facility (See Supplementary Fig. 12 for Gating strategy).

### Gas6 ELISA

WT endothelial cell, B16F0 cell and WT and FAK^KO^ pericyte conditioned medium (CM) was collected and centrifuged to remove cell debris, prior to short term storage at 4 °C. Gas6 levels were measured in CM using the Gas6 ELISA (ThermoFisher Scientific) according to the manufacturers’ instructions.

### B16 and CRISPR/Cas9-KO cell tumour growth

WT C57/Bl6 mice were given a single subcutaneous injection into the flank of 1 × 10^5^ B16F0 cells mixed with 8 × 10^5^ CRISPR/Cas9-KO pericytes. For B16 CRISPR/Cas9-KO experiments, 1 × 10^6^ B16F0 CRISPR/Cas9-KO cells were injected into either *pdgfrßcre*+;*fakfl/fl* and *pdgfrßcre-;fakfl/fl* mice. Note, FAK-nullCas9 and WTCas9 tumour growth and blood vessel density controls are identical in Fig 3e, h, Fig 4e and supplementary fig 10 because they were common controls from the same experimental run.

### Immunostaining

Five micrometer frozen tumour sections were air-dried for 10 min, washed once in PBS, fixed in acetone for 10 min at −20 °C, washed in PBS three times and then blocked with 5% normal goat serum for 30 min at room temperature. After blocking, sections were incubated with primary antibodies overnight at 4 °C. Primary antibodies used were directed against endomucin (V.7C7, 1:100), NG2 (AB5320, Millipore, 1:100), α-SMA (A2547, Sigma, 1:100), Ki67 (ab15580, Abcam, 1:100). Sections were then washed with PBS and incubated with Alexa-Fluor^®^-conjugated secondary antibodies (Invitrogen) for 45 min at room temperature before mounting the slides with Prolong^®^ Gold anti-fade reagent (Invitrogen). Formaldehyde fixed sections were de-waxed by immersion in xylene for 5 and 3 min, followed by re-hydration in a gradient of ethanol diluted in distilled water (100, 90, 70, and 50%) for 5 min in each solution. After washing once in PBS, antigen retrieval was performed by heating the samples in 10 mM Na citrate buffer (trisodium citrate diluted in distilled water, pH 6.0) for 20 min in a microwave. The samples were then incubated for 5 min at room temperature in 3% hydrogen peroxide diluted in methanol. Endomucin or SV40 (Pab101, sc-147, Santa Cruz) antibodies, diluted 1:200 in 1% normal goat serum (NGS) and PBS, were incubated on the tissue sections overnight at 4 °C. After incubation, tissue sections were washed three times in PBS followed by 1 h incubation at room temperature with the appropriate secondary biotinylated antibodies (Dako), diluted 1:100 in 1% NGS PBS. After washing with PBS the tissue sections were incubated with the ABC reagent (PK-6102, Vector) for 30 min, washed again and DAB substrate (SK-4100, Vector) was added for 5–10 min until sections turned brown. After washing, the sections were counterstained with Hematoxylin/Eosin, dehydrated and mounted in DPX. A Zeiss AxioPlan microscope and AxioVision software were used for imaging the slides. For FAK immunostaining (3285, Cell Signalling, 1:100), after incubation with primary antibody, slides were incubated with swine anti-rabbit biotin (E0353, DAKO, 1:100) followed by incubation with streptavidin-HRP (FP1047, TSA Fluorescence Systems) for 30 min then fluorescein (FP1018, TSA Fluorescence Systems) for 10 min.

For quantification of pericyte coverage, tumour sections were immunostained with endomucin (1:100) and α-SMA (1:100) and the numbers of endomucin+/α-SMA+ and endomucin+/α-SMA- blood vessel were counted. From this the % of endomucin+/α-SMA+ positive blood vessels were calculated.

For PDGFR*β* staining and immune cell infiltration staining see Supplementary Methods.

### Ex vivo aortic ring assay

Thoracic aortas were isolated and prepared for culture as previously described^48^. Briefly, 0.5 mm thick rings were serum starved for 18 h at 37 °C before being embedded in 1 mg/ml rat tail collagen type I (Millipore) and cultured in Opti-MEM^®^ (Gibco) supplemented with 2.5% foetal calf serum (FCS) with either PBS as a control or 100 ng/ml Gas6 (R&D). Sprouting microvessels were counted as specified in the figure legends. Following fixation in 4% PFA, the rings were stained with FITC-conjugated BS-1 Lectin (L9381, Sigma, 1:200) then mounted on slides using ProLong-Gold^TM^ with anti-fade reagent (Invitrogen). Zeiss AxioPlan microscope and AxioVision software were used for imaging.

### Subcutaneous angiogenesis assay

Two autoclaved synthetic sponges (a kind gift from Daryl Harmon, Caligen Foam Ltd.) of approximately 1 cm^2^ were implanted subcutaneously in both flanks of anaesthetised mice. Every two days, sponges were injected with either 100 µl of PBS alone or 100 µl of PBS containing 10 ng/ml VEGF, 10 ng/ml PDGF-B or 10 ng/ml PlGF (Peprotech). After 14 days, sponges were excised and PFA fixed for paraffin embedding. Sections of sponges were immunostained for endomucin (1:100) to identify blood vessels, and density was assessed by counting the numbers of endomucin-positive blood vessels/area of sponge section.

### HUVEC bead sprouting assay

650 HUVECs were seeded in drops of 20 μl of medium containing 0.25% of methylcellulose (Sigma-Aldrich) and left overnight to form spheroids by the “hanging drop” method. Next day, the spheroids were embedded in 1 mg/ml collagen gels, and then stimulated with Optimem + 1% FBS supplemented with PBS or 100 ng/ml Cyr61. After 24 h, gels were fixed in 2% PFA, stained with Rhodamine Phalloidin (R415, ThermoFisher, 1:1000) and cumulative length of all sprouts from each spheroid were quantified.

### Primary cell cultures

Primary mouse lung ECs and primary mouse brain pericytes were isolated from *pdgfrβcre*+;*fakfl/fl* and *pdgfrβcre-;fakfl/fl* control mice and cultured as previously described^49,50^. For endothelial cells, *pdgfrβcre*+;*fakfl/fl* and *pdgfrβcre-;fakfl/fl* mouse lungs were minced, collagenase digested (Type I, Gibco), strained through a 70 μm cell strainer (BD Falcon) and the resulting cell suspension plated on flasks coated with a mixture of 0.1% gelatin (Sigma), 10 μg/ml fibronectin (Millipore) and 30μg/ml rat tail collagen (Sigma). Endothelial cells were purified by a single negative (FCγ-RII/III; Millipore, MABF838) and two positive cell sorts (ICAM-2; Pharmingen, 553326), using anti-rat IgG-conjugated magnetic beads (Dynal). During preparation of primary endothelial cells, lung fibroblasts were isolated from the non-endothelial cell population that was generated during the first positive sort. For all cell types, passaging occurred when cells reached 70% confluency. Cells were trypsinised, centrifuged, washed with PBS and replated on pre-coated flasks for endothelial cells and pericytes and non-coated flasks for fibroblasts. Fibroblasts were cultured in DMEM+ 10% FCS to passage 4, Endothelial cells in MLEC (Ham’s F-12, DMEM (low glucose), 10% FCS, heparin and endothelial mitogen (Generon) to passage 4–5. Briefly, for pericytes, brains were removed from mice, minced, digested for 1 h in 0.1% collagenase, centrifuged in the presence of 22% BSA, and cultured in endothelial cell growth media (pMLEC) with the medium changed every 3 days. On reaching confluency, cultures were harvested with trypsin and passaged. During the first two passages, pericyte cultures were grown in pMLEC, and on the third passage they were grown in pericyte medium (PM; ScienCell Research Laboratories) containing 2% FBS and antibiotics. Tissue culture plates for all experiments were coated with a mixture of collagen (30 µg/ml), gelatin (0.1%) and fibronectin (10 µg/ml). Primary mouse brain pericytes were characterised using the following markers, by FACS: PDGFR*β*, CD146, CD31 (endothelial marker), glial fibrillary acidic protein and Mac1 (fibroblast markers). Primary mouse lung endothelial cells were stained for CD31 followed by FACS analysis and immunostained for endomucin and vimentin (expressed by ECs) and α-sma (fibroblast marker). Primary mouse lung fibroblasts were immunostained for vimentin (positive marker for fibroblasts), NG2 and endomucin (negative markers for fibroblasts), as previously described^51^.

### Growth factor stimulation and Western blot analysis

WT and FAK^KO^ pericytes at around 70% confluence were serum starved for 24 h in Optimem then treated with PDGF-B (30 ng/ml) (Peprotech) or Gas6 (100 ng/ml or 500 ng/ml) (R&D) for 0, 5, 15, 30, and 60 min. ECs at around 80% confluence were serum starved for 24 h then treated with either VEGF (30 ng/ml) or PlGF (30 ng/ml) (Peprotech) for 0, 5, 15 30, and 60 min. For PI3K inhibitor experiments, after 24 h serum starvation, 1 μM PI3K inhibitor (GDC-0941) was incubated in the culture medium for 24 h prior to stimulation with Gas6. For both pericytes and ECs, lysates were prepared in RIPA buffer containing protease and phosphatase inhibitors (Roche). For analysis of protein levels, cells were subjected to SDS-PAGE and transferred to nitrocellulose membranes (Amersham Biosciences) for Western blotting. Blots were probed for phospho- and total PDGFRß (3161, 3169, Cell Signalling, 1:1000), phospho and total ERK1/2 (9101, 9102, Cell Signalling, 1:1000), phospho-and total AKT (4685, 9611, Cell Signalling, 1:1000), AKT 1 (2967, Cell Signalling, 1:1000), phospho- and total SAPK/JNK (9251, 9252, Cell Signalling, 1:1000), phospho- and total VEGFR2 (9698, 2478, Cell Signalling, 1:1000), phospho-p65 and total p65 (3033, 8242, Cell Signalling, 1:1000), p-Axl (E-AB-34244, Elabscience, 1:5000), phospho-Tyro3 (PA5-40270, ThermoFisher, 1:2000) and Tyro3 (5585, Cell Signalling, 1:1000), Cyr61 (ABC102, Millipore, 5 μg/ml), Pyk2 and p-Pyk2 (3090, 3291, Cell Signalling, 1:1000), Src and p-Src (2108, 6943, Cell Signalling, 1:1000), Tubulin (T5168, Sigma, 1:5000), GAPDH (AB2302, Millipore, 1:5000) and HSC-70 (Santa Cruz, sc-7298, 1:5000-1:10,000). Densitometric readings of band intensities were obtained using the ImageJ^TM^ software (see Supplementary Fig. 13 for uncropped scans).

### p-Axl immunostaining of pericytes

FAK-null;Gas6^KO^ and FAK-null;Cas9 control pericytes were seeded on glass coverslips for 24 h. Cells were washed with PBS and fixed with 4 % PFA for 15 min at room temp and washed again. Cells were permeabilised with 0.4% TritonX-100 in PBS for 15 min at room temp and washed with PBS, followed by blocking for 20 min (2.5% goat serum+ 1% BSA+ 0.1% PBS-T). Cells were incubated overnight at 4 °C with p-Axl primary antibody (R&D, AF2228) diluted 1:100 in blocking solution then washed three times with PBS followed by incubation with Alexa Fluor goat anti-rabbit 488 (Invitrogen, A32731, 1:500 dilution in blocking solution) for 1 h at room temp. Cells were washed three times with PBS, once with water and mounted with Prolong Gold + DAPI (Invitrogen).

### Protein arrays

WT and FAK^KO^ pericyte angiogenesis profiles were compared using the angiogenesis array (ARY015, R&D Biosystems) and phospho-RTK profiles were compared using the phospho-RTK array (ARY014, R&D Biosystems). Briefly, cell lysates were prepared as follows: sample buffer was added to the cell culture, the cells were scraped and transferred into a 1.5 ml Eppendorf tube. After sonication, samples were adjusted to the array conditions and mixed with a Detection Antibody Cocktail as indicated by the manufacturer’s instructions. Lysates were incubated overnight at 4 °C on dot-blot membranes. Membranes were washed, incubated with streptavidin-HRP for 30 min at RT, washed again and ECL was applied to the membrane to reveal the dots.

WT or FAK^KO^ pericytes were co-cultured with B16F0 tumour cells at a ratio of 5:1 in pericyte medium. After 24 h, cells were separated by FACS using the PDGFR*β* receptor antibody (NBP1-43349, Novus Biological, 1:100) which is positive for pericytes but not for B16F0 tumour cells. Once sorted, the cells were lysed in sample buffer and analysed using the angiogenesis array as described above. For the phospho-RTK arrays, WT and FAK^KO^ pericytes were then lysed as described above and analysed using the phospho-RTK array. Densitometric analysis was performed using ImageJ^TM^ software. Briefly, the same row of array from multiple experiments, at the same exposure time, were combined and densitometric analysis performed.

### Adhesion assay

WT and FAK^KO^ pericyte adhesion properties were measured using the CytoSelect™ 48-Well Cell Adhesion Assay (CBA-070, Cell Biolabs). Detached cells (25,000 cells/well) were seeded on each matrix for 45 min, washed and fixed following the manufacturer’s instructions. Cell Stain solution was applied and OD measured at 560 nm with a plate reader.

### Proliferation assay

WT and FAK^KO^ pericytes were seeded in a 24-well plate and subjected to images every 6 h using the IncuCyte® ZOOM system (Essen Bioscience). Confluency was quantified by analysing cell images over time, using IncuCyte® ZOOM software (Essen Bioscience). Cell confluency was expressed as the percentage of coverage of the microscopic field of view.

### Dunn chamber assay

Random migration was analysed using Dunn Chambers. Dunn Chambers were stored in 7× detergent, washed in water to remove any wax, rinsed in distilled water and sterilised in ethanol before use. Chambers were rinsed in media and both outer and inner wells were filled with OPTI-MEM. WT and FAK^KO^pericytes were plated on coated 22 mm glass coverslips and cultured overnight. The following day cells were washed with PBS and serum starved for 3 h in serum-free medium (OPTI-MEM I + GlutaMAX, Gibco, Invitrogen, Paisley, UK). Coverslips were inverted onto the Dunn Chambers leaving a gap in the outer well and sealed on three sides with hot wax mixture (Vaseline:paraffin:beeswax—1:1:1). The media was removed from the outer well by capillary action and was rinsed with OPTI-MEM before filling with pericyte medium. The chamber was then sealed with wax and mounted on a Zeiss Axio100 inverted microscope. Images were acquired by phase contrast imaging using a 10× N-Achroplan Phase contrast objective (NA 0.25). Cell images were collected using a Sensicam (PCO Cook) CCD camera, taking a frame every 10 min for 16 h using Micro Manager acquisition software (NIH, open source). Subsequently all the acquired time-lapse sequences were displayed as an AVI file and cells from the time-lapse sequence were tracked using ImageJ. Tracking resulted in the generation of a sequence of position coordinates relating to each cell in each frame, motion analysis was then performed on these sequences using Wolfram Mathematica 7 software.

### RT-qPCR

RNA was extracted from cells using the RNeasy mini kit according to the manufacturer’s instructions, (Qiagen UK, Manchester, UK). Complementary DNA (cDNA) synthesis was carried out using the Applied Biosystems High Capacity cDNA Reverse Transcription (RT) Kit (Fisher Scientific) as per the manufacturer’s instructions. Five hundred nanogram of mouse cell cDNA was used for RT-qPCR. RT-qPCR was performed using sample cDNA (FAM-tagged), an internal control *Gapdh* (VIC-tagged) and specific TaqMan probes (*Axl, Gas6, Fak, Pdgfrβ*). qPCR was carried out using the TaqMan Universal PCR Master Mix (PE Applied Biosystems, Fisher Scientific) in a 96-well plate. Hundred and sixty nanogram of cDNA from each sample was amplified using qPCR across 40 cycles. Target mRNA was normalised to *Gapdh*, and the expression level of each gene determined relative to the initial experimental controls using the 2^−ΔΔCT^ method.

### Human tissue sections

Formalin fixed paraffin embedded tissue samples from human melanoma were sectioned, dewaxed, and antigen retrieval carried out in boiling 10 mM citrate buffer pH 6.0, 5 mm sections were washed three times in PBS, blocked in 1% normal goat serum (NGS) 0.1% TritonX-100 (TX-100) for 1 h. Sections were double immunostained for mouse monoclonal anti-alpha-smooth muscle actin Cy3-conjugated (Sigma-Aldrich, C6198, 1:100) and for FAK (3285, Cell Signalling, 1:100). Staining was performed as described in the Immunostaining section. For data analysis, the percentage of FAK and α-SMA-double-positive blood vessels was calculated as the number of FAK and α-SMA-double-positive blood vessels over the total number of α-SMA-positive blood vessels. Patient tissue samples with less than 50% of α -SMA-positive blood vessels with FAK expression were classified as low number of mural FAK- positive blood vessels.

### Statistical analysis and reproducibility

The statistical significance of differences among mean values was determined by one-way ANOVA analysis and unpaired two-sided Students *t*-test, *p* < 0.05 was considered statistically significant, unless otherwise indicated. For tumour growth statistics, non-parametric Mann–Whitney *U* rank sum test was performed to compare tumour volumes each day.

### Ethical regulations

All procedures were approved by our local animal ethics committee, Queen Mary University of London, and were executed in accordance with United Kingdom Home Office regulations.

Human melanoma samples were obtained with signed informed consent from patients and ethical committee approval. Melanoma samples were covered by East London and City Health Authority Ethical Approval (07/Q0604/23 MM: East London and City Health Authority: “Molecular mechanisms in the pathogenesis of malignant melanoma”), with a minor amendment in March 2018: ReDa 005044Q1.

### Reporting summary

Further information on research design is available in the Nature Research Reporting Summary linked to this article.

## Supplementary information

Supplementary Information

Reporting Summary

## Data Availability

All the relevant data that support the findings of this study are available from the corresponding author on request. Source data are provided with this paper.

## Source data

Source Data
