# Author Correction: Pericyte FAK negatively regulates Gas6/Axl signalling to suppress tumour angiogenesis and tumour growth

**DOI:** 10.1038/s41467-023-41239-0

**Published:** 2023-09-06

**Authors:** Tanguy Lechertier, Louise E. Reynolds, Hyojin Kim, Ana Rita Pedrosa, Jesús Gómez-Escudero, José M. Muñoz-Félix, Silvia Batista, Matthew Dukinfield, Fevzi Demircioglu, Ping Pui Wong, Kylie P. Matchett, Neil C. Henderson, Gabriela D’Amico, Maddy Parsons, Catherine Harwood, Pascal Meier, Kairbaan M. Hodivala-Dilke

**Affiliations:** 1 Centre for Tumour Biology, Barts Cancer Institute – a CR-UK Centre of Excellence, Queen Mary University of London, John Vane Science Centre, Charterhouse Square, London, EC1M 6BQ UK; 2 Cell Death & Inflammation, The Breast Cancer Now Toby Robins Research Centre, Institute of Cancer Research, Fulham Road, London, SW3 6JB UK; 3 Systems Oncology Group, Champalimaud Research, Champalimaud Centre for the Unknown Av. Brasília, Doca de Pedrouços, 1400-038 Lisbon, Portugal; 4 Present Address: Guangdong Provincial Key Laboratory of Malignant Tumor Epigenetics and Gene Regulation, Medical Research Center, Sun Yat-Sen Memorial Hospital, Sun Yat-Sen University, 510120 Guangzhou, China; 5 Centre for Inflammation Research, The Queen’s Medical Research Institute, Edinburgh BioQuarter, University of Edinburgh, Edinburgh, UK; 6 MRC Human Genetics Unit, Institute of Genetics and Molecular Medicine, University of Edinburgh, Crewe Road South, Edinburgh, UK; 7 Nikon Imaging Centre@King’s, Randall Division of Cell and Molecular Biophysics, Kings College London, Room 3.22B, New Hunts House Guys Campus, London, SE1 1UL UK; 8 Centre for Cell Biology and Cutaneous Research, Blizard Institute, Barts and The London School of Medicine and Dentistry, Queen Mary University of London, London, E1 2AT UK

**Keywords:** Growth factor signalling, Cancer microenvironment

Correction to: *Nature Communications* 10.1038/s41467-020-16618-6, published online 04 June 2020

The original version of this Article contained errors in Supplementary Fig. 1d in which the H&E stained sections of lung, heart and liver from the mice were incorrect. These errors have been corrected in the HTML and PDF versions of the Article.

### Supplementary information

Updated Supplementary Information

