# Supplementary material for: Pericyte FAK negatively regulates Gas6/Axl signalling to suppress tumour angiogenesis and tumour growth
Source: Nat Commun. 2020 Jun 4;11:2810. doi: 10.1038/s41467-020-16618-6 (PMC7272651; doi:10.1038/s41467-020-16618-6)
Supplement: Supplementary file 1 — Supplementary Information [file 41467_2020_16618_MOESM1_ESM.pdf]

Pericyte FAK negatively regulates Gas6/Axl signalling to suppress tumour angiogenesis and tumour growth

Lechertier *et al*.

Supplementary Figure 1

Lechertier et *al.*

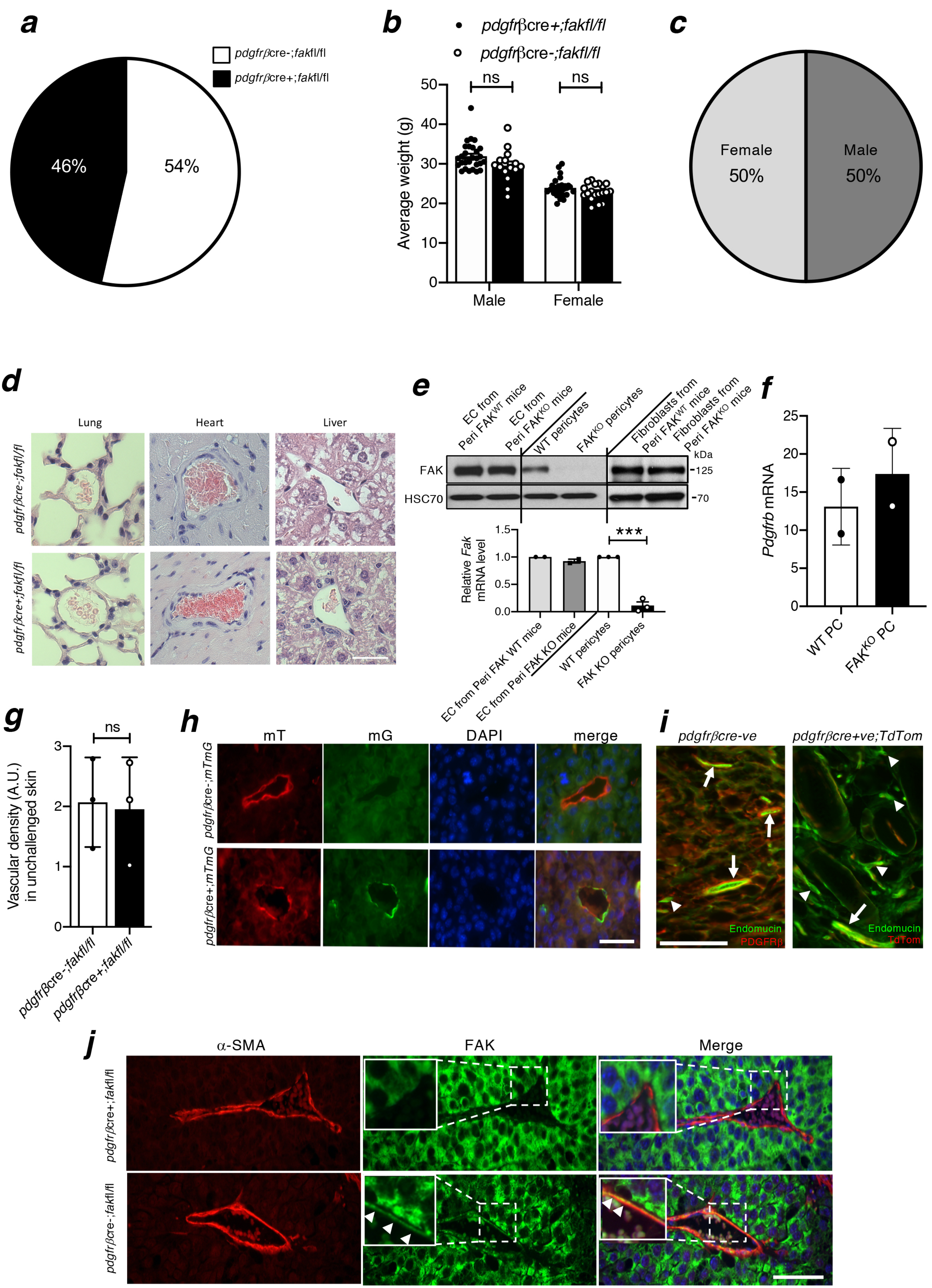

Supplementary Figure 1: **Characterisation of pericyte FAK-deficient mice.** (**a**) Normal Mendelian ratios of *pdgfrβcre+;fakfl/fl* and *pdgfrβcre-;fakfl/fl* mice. n=100 *pdgfrβcre+;fakfl/fl* and 100 *pdgfrβcre-;fakfl/fl* mice. (**b**) Post-weaning weights of *pdgfrβcre-;fakfl/fl* and *pdgfrβcre+;fakfl/fl* mice are similar, n=50 *pdgfrβcre+;fakfl/fl* and 50 *pdgfrβcre-;fakfl/fl* mice. Bar chart shows mean weight (g)±s.e.m for *pdgfrβcre-;fakfl/fl* (white bars) and *pdgfrβcre+;fakfl/fl* (black bars), ns=not significant. Two-sided Students *t* test. **(c)** Male to female ratios were normal in *pdgfrβ* cre+;*fakfl/fl* mice; n=100 mice. **(d)** Representative H&E stained sections of lung, heart, liver and spleen from 12 week old *pdgfrβcre+;fakfl/fl* and *pdgfrβcre-;fakfl/fl* mice. No gross morphological defects in large blood vessels were observed; n=3 tissue sections/genotype. Scale bar, 100 µm. (**e**) Western blotting for FAK in endothelial cells (EC), pericytes and fibroblast from *pdgfrβcre-;fakfl/fl* and *pdgfrβcre+;fakfl/fl* lysates. FAK protein and mRNA levels were significantly reduced only in pericytes from *pdgfrβcre+;fakfl/fl* mice and not in fibroblasts or ECs. n=1 (fibroblasts) and 3 (EC and PC) experimental repeats for WBs. Bar chart represents mean relative *Fak* mRNA level±s.e.m., n=3 experimental repeats. ***p=0.0002. Two-tailed Students *t* test. (**f**) mRNA levels of *Pdgfrb* were unchanged between the two genotypes. Bar chart represents mean relative *Pdgfrb* mRNA levels±s.d., n=2 experimental repeats. Two-sided Students *t* test. **(g)** Vascular density from unchallenged skin is unchanged in *pdgfrβcre+;fakfl/fl* mice. Bar chart represents the mean vascular density±s.e.m. n=3 skin sections/genotype, ns=not significant. Two-sided Students *t* test. (**h**) *Pdgfrβcre-* and *pdgfrβcre+* mice were crossed with *mTmG* reporter mice, producing both *pdgfrβcre-;mTmG* and *pdgfrβcre+;mTmG* mice. Mice were injected subcutaneously with B16F0 cells. Sections displayed both membrane-targeted tandem dimer Tomato (mT) (red), seen in all host tissues. The membrane-targeted green fluorescent protein (GFP) (mG) (green), which is observed after Cre excision was found in *pdgfrβcre+;mTmG* mice only, as expected and exclusively in pericytes of tumour blood vessels; n=3 sections/genotype. Scale bar, 50 µm. **(i)** Unchallenged adult skin from *pdgfrβ*cre-;TdTom mice. PDGFRβ is weakly expressed and only in 76 % dermal vessels. TdTom reporter signal was found in only 32.6 % of dermal blood vessels of *pdgfrβcre+;*TdTom mouse unchallenged skin. The results indicate that *Pdgfrβ* promotor activity is very poor in quiescent unchallenged skin vessels. N= 3 sections/genotype. Arrows, PDGFRβ positive vessels; arrowheads, PDGFRβ negative vessels. Scale bar, 10 µm. **(j)** Immunofluorescence showing pericyte FAK-loss in tumour blood vessels stained for FAK and α-SMA from *pdgfrβcre+;fakfl/fl* but not *pdgfrβcre-;fakfl/fl* mice. N= 2 tumour sections/genotype. Inset, *arrow head*, pericyte FAK staining; scale bar, 20 µm. Source data are provided as a Source data file.

Supplementary Figure 2

Lechertier *et al.*

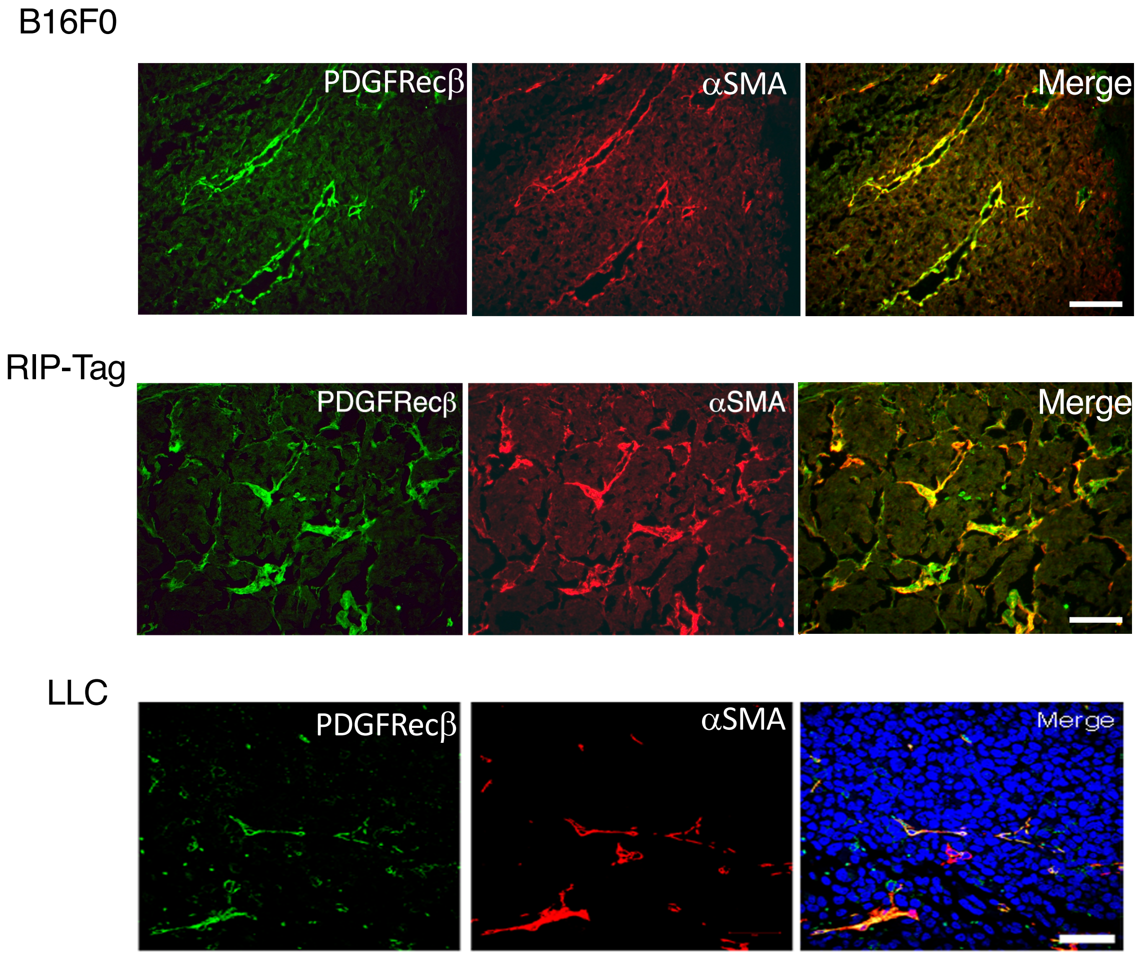

***Supplementary Figure 2*: PDGFRβ expression is limited to pericytes in B16F0, RIP-Tag and LLC mouse tumours.** PDGFRβ immunostaining was performed on B16F0, LLC and RIP-Tag tumour sections. Double immunostaining with α-SMA confirmed that PDGFRβ was localised to pericyte markers and staining was not observed in the tumour stroma. N= 6 tumours/genotype. Scale bar, 100 μm. Source data are provided as a Source data file.

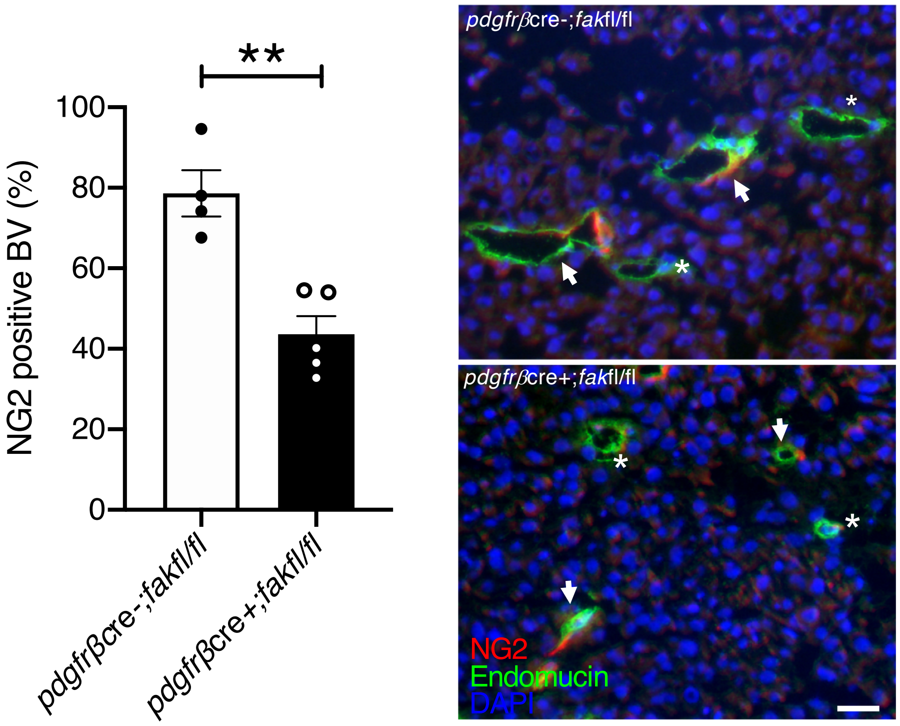

***Supplementary Figure 3*: Reduced percentage of NG2-positive pericyte association with blood vessels in *pdgfrβcre+;fakfl/fl* mice.** NG2-positive pericyte association with tumour blood vessels was assessed by double immunofluorescence staining of midline tumour sections for endomucin (green) and NG2 (red). DAPI (blue) was used as a nuclear counterstain. Numbers of tumour blood vessels with associated pericytes are reduced in *pdgfrβcre+;fakfl/fl* mice. Bar charts represent the percentage of NG2 positive blood vessels±s.e.m. n=4 tumours grown in *pdgfrβcre-;fakfl/fl*, 5 tumours grown in *pdgfrβcre+;fakfl/fl*, scale bar, 50 µm.** p=0.0018. Two-sided Students *t* test. Source data are provided as a Source data file.

a

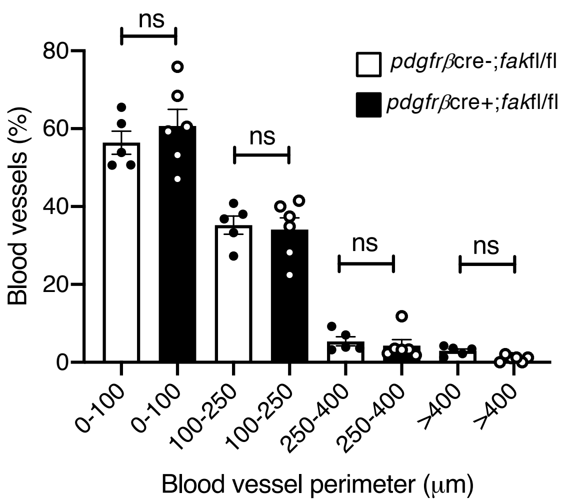

b

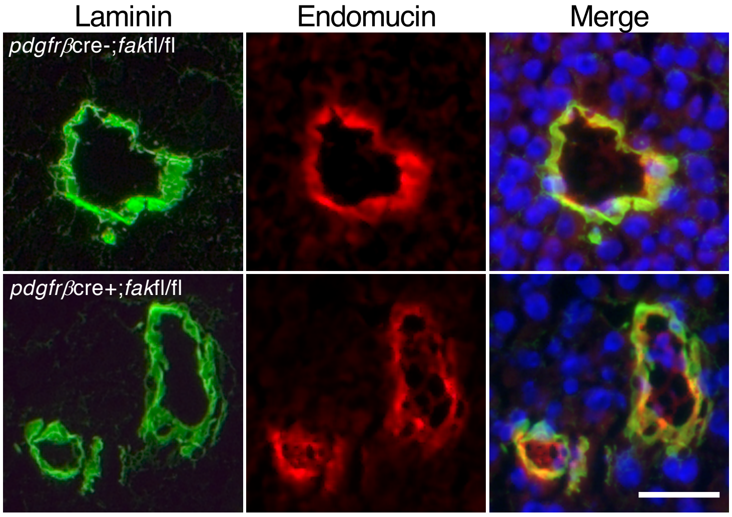

c

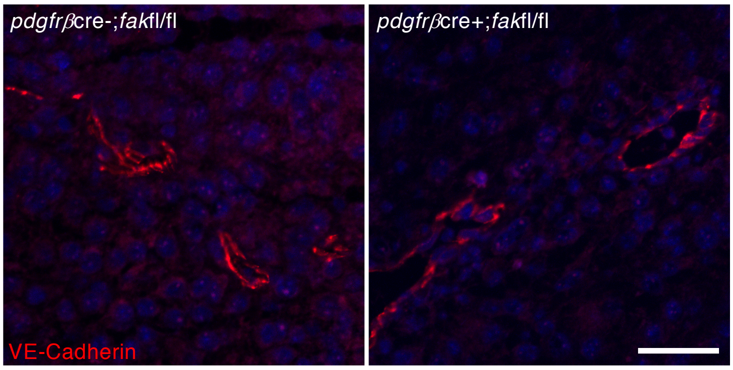

Supplementary Figure 4: **Tumour microvessel dilation and laminin deposition are not affected by the loss of pericyte FAK. (a)** The perimeters of endomucin-positive microvessels were measured in sections of tumour grown in *pdgfrβcre-;fakfl/fl* and *pdgfrβcre+;fakfl/fl* mice. No significant difference between genotypes was observed. Bar chart represents mean no. blood vessels (%)±s.e.m., n=382 blood vessels in tumours from *pdgfrβcre-;fakfl/fl* mice and n=703 blood vessels in tumours from *pdgfrβcre+;fakfl/fl* mice. Two-sided Students *t* test. (**b**) Immunostaining of tumour blood vessels for laminin and endomucin showed no obvious defects in laminin deposition or intensity. N=3 tumours/genotype. Scale bar, 50 µm. (**c**) VE-cadherin staining of endothelial cells in blood vessels showed no differences between tumours grown in *pdgfrβcre-;fakfl/fl* and *pdgfrβcre+;fakfl/fl* mice; n= 3 tumours/genotype. Scale bar, 50 µm. Source data are provided as a Source data file.

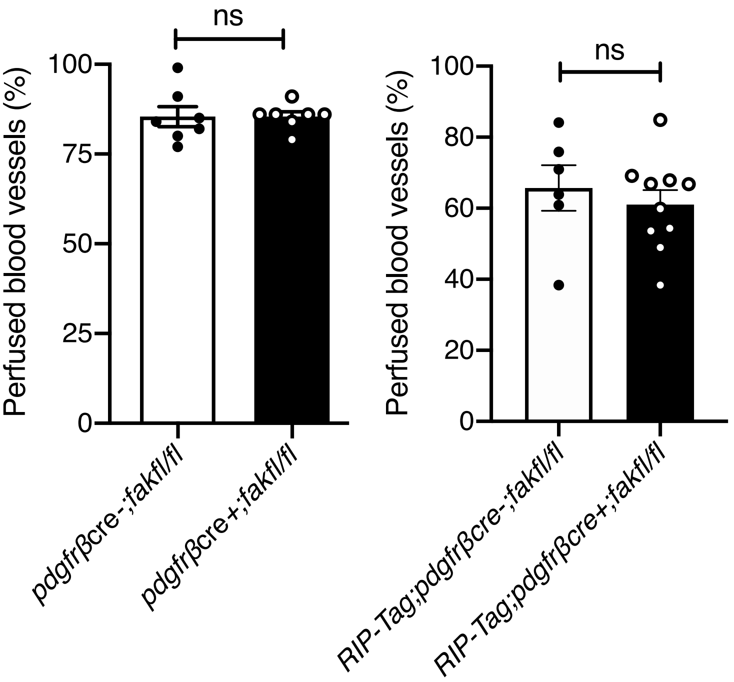

Supplementary Figure 5: **Tumour blood vessel perfusion is not affected by the loss of pericyte FAK.** Blood vessel perfusion was quantified from B16F0 tumours grown in *pdgfrβcre-;fakfl/fl* and *pdgfrβcre+;fakfl/fl* and *RIP-Tag;pdgfrβcre-;fakfl/fl* and *RIP-Tag;pdgfrβcre+;fakfl/fl* mice. No significant difference in blood vessel perfusion between the genotypes of both models was observed. Bar chart represents the % of perfused blood vessels in tumour sections±s.e.m. n=7 tumours grown in *pdgfrβcre-;fakfl/fl*, 7 tumours grown in *pdgfrβcre+;fakfl/fl*; n=6 tumours grown in *RIP-Tag;pdgfrβcre-;fakfl/fl*, 10 tumours grown in *RIP-Tag;pdgfrβcre+;fakfl/fl* mice; ns= not significant; two-sided Students *t* test. Source data are provided as a Source data file.

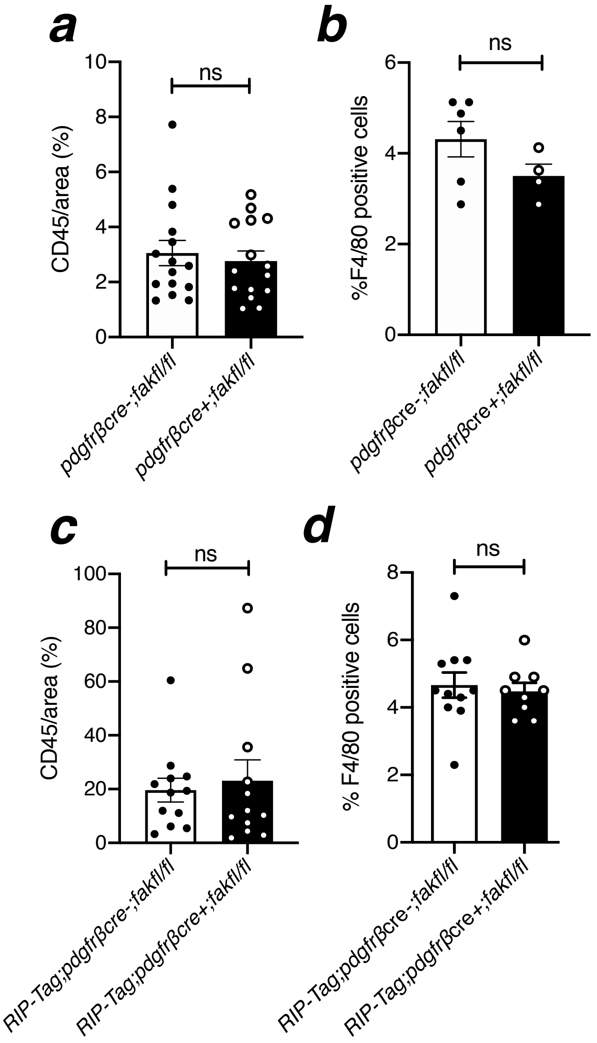

Supplementary Figure 6: **Immune infiltration of CD45- and F4/80-positive cells is similar in B16F0 tumours grown in *pdgfrβcre-;fakfl/fl*, *pdgfrβcre+;fakfl/fl* and pancreatic insulinomas in RIP-Tag; *pdgfrβcre-;fakfl/fl* and RIP-Tag; *pdgfrβcre+;fakfl/fl* mice.** Sections of B16F0 tumours from *pdgfrβcre-;fakfl/fl* and *pdgfrβcre+;fakfl/fl* mice were immunostained for **(a)** CD45-positive leukocytes and **(b)** F4/80-positive macrophages. Sections of insulinomas from *RIP-Tag;pdgfrβcre-;fakfl/fl* and *RIP-Tag;pdgfrβcre+;fakfl/fl* mice were immunostained for **(c)** CD45-positive leukocytes and **(d)** F4/80-positive macrophages. The area of immune infiltrate signal was determined histologically and presented as percentage of total tumour section area. Bar charts represents % mean of inflammation area±s.e.m.; n=5 tumours grown in *pdgfrβcre-;fakfl/fl*, 5 tumours grown in *pdgfrβcre+;fakfl/fl*, 11 tumours grown in RIP-Tag; *pdgfrβcre-;fakfl/fl*, 9 tumours grown in RIP-Tag; *pdgfrβcre+;fakfl/fl* mice. ns= not significant. Two-sided Students *t* test. Source data are provided as a Source data file.

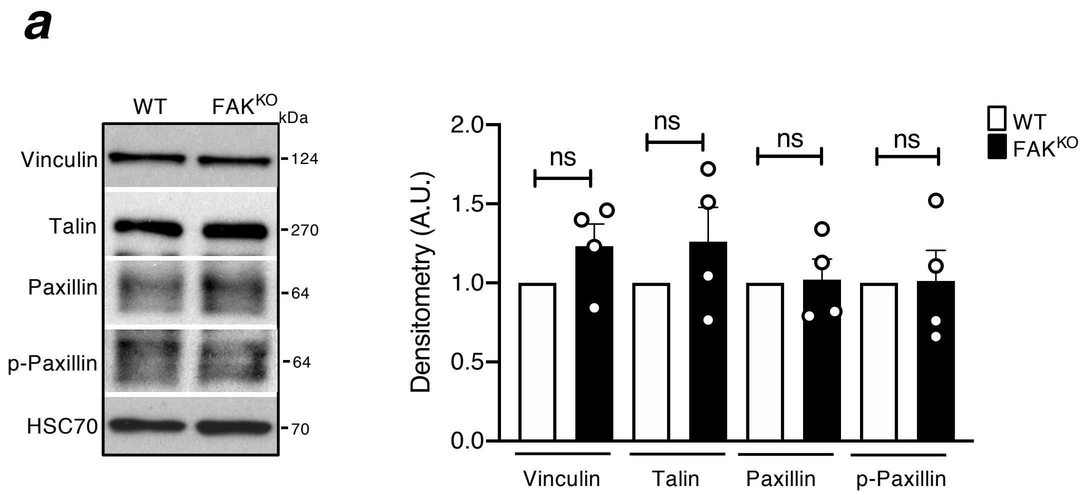

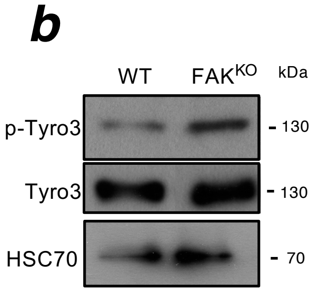

Supplementary Figure 7: **Focal contact protein expression is not altered in FAK^{KO} pericytes. (a)** Western blot analysis of focal contact proteins in WT and FAK^{KO} pericytes. Western blot analysis determined the expression levels of Vinculin, Talin, Paxillin and phospho-Paxillin were similar in both genotypes. HSC70 acts as a loading control. Bar chart represents the densitometric quantitation±s.e.m. n= 4 experimental repeats. ns= not significant. Two-sided Students *t* test. **(b)** p-Tyro3 levels were increased in FAK^{KO} pericytes compared with WT pericytes. HSC70 acts as a loading control. N=2 experimental repeats. Source data are provided as a Source data file.

Supplementary Figure 8

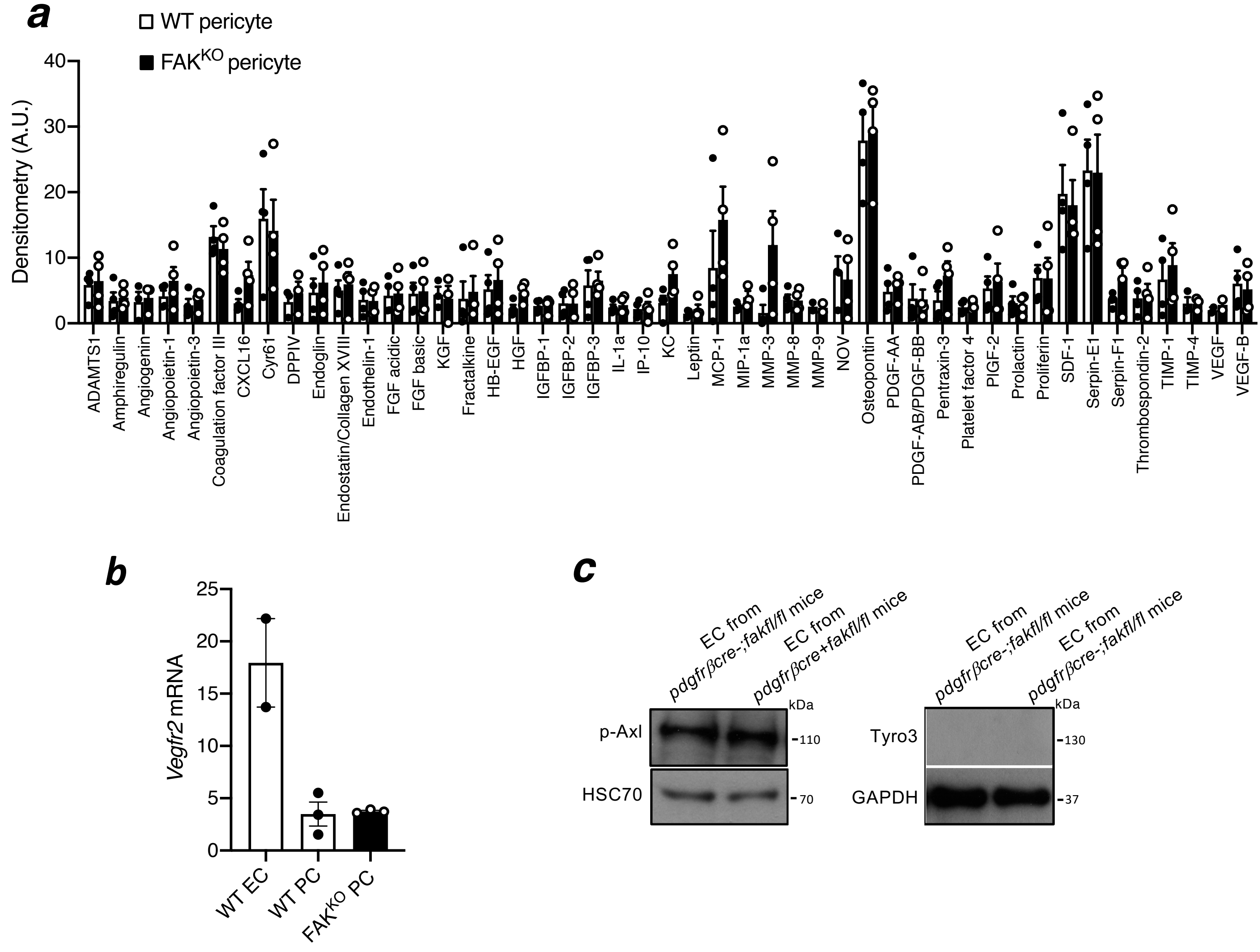

Supplementary Figure 8: **No changes in angiogenesis related protein or *Vegfr2* levels in unstimulated WT and FAK-null pericytes. No changes in pAxl or Tyro3 in ECs from *pdgfrβcre-;fakfl/fl* and *pdgfrβcre+;fakfl/fl* mice.** **(a)** Quantitation of WT and FAK^{KO} pericyte angiogenesis regulators protein expression profiles show no significant difference in protein expression between the two genotypes. Bar chart represents mean densitometric reading of Angiogenesis protein profiler WT (white bars) or FAK^{KO} (black bars)±s.e.m., n=4 experimental repeats. **(b)** *Vegfr2* mRNA levels were almost undetectable in WT and FAK^{KO} pericytes, compared with WT endothelial cells. Bar chart represents mean relative *Vegfr2* mRNA levels±s.e.m., n=3 experimental repeats, ns=not significant. Two-sided Students *t* test. **(c)** Western blot analysis of EC lysates from *pdgfrβcre-;fakfl/fl* mice and *pdgfrβcre+;fakfl/fl* mice show no change in p-Axl levels and no expression of Tyro3. HSC70 and GAPDH act as loading controls, n=3 experimental repeats. Source data are provided as a Source data file.

Supplementary Figure 9

Lechertier *et al.*

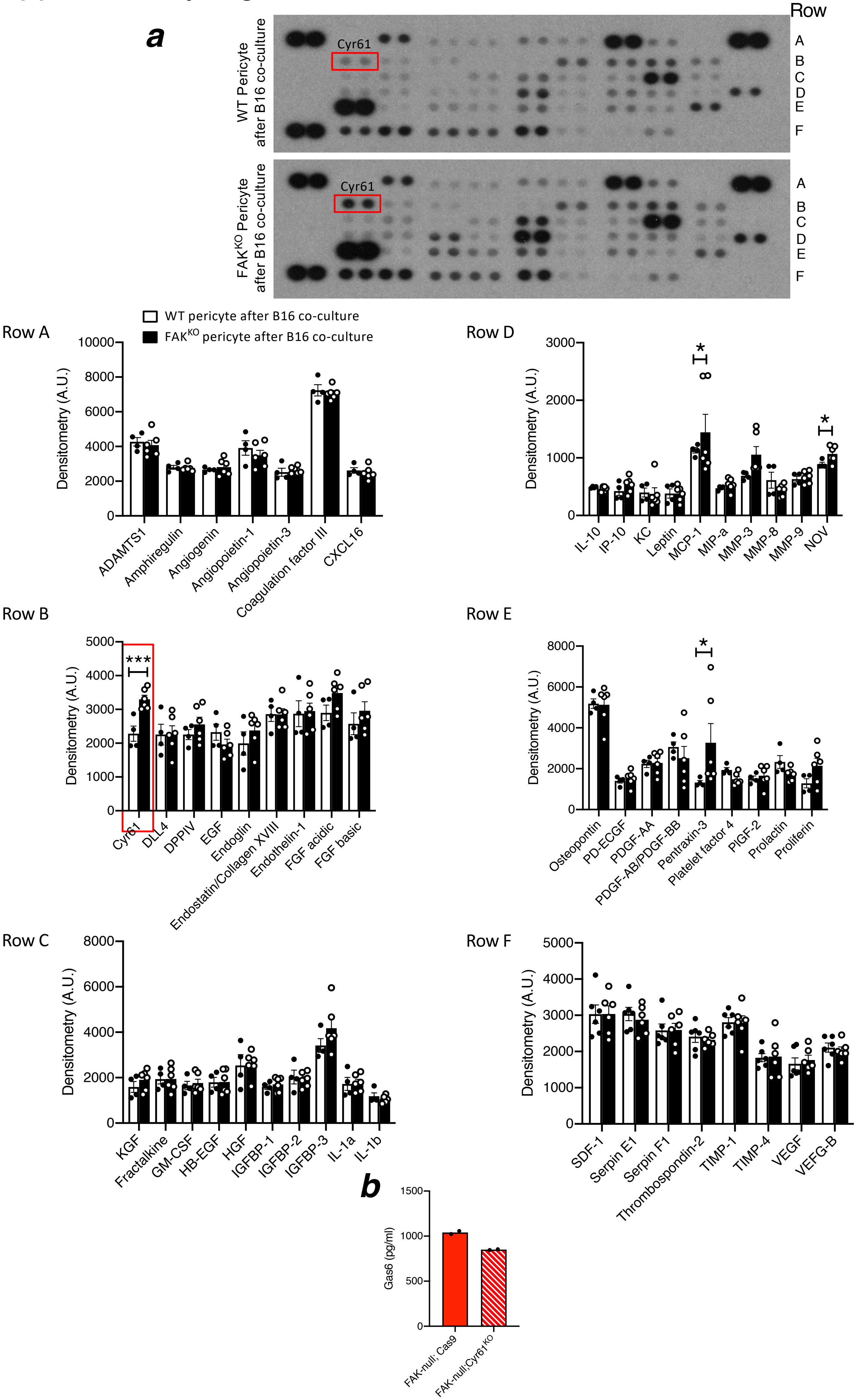

Supplementary Figure 9: **Angiogenesis protein expression dot blot array results from WT and FAK^{KO} pericytes after co-culture with B16F0 tumour cells. (a)** Representative arrays and quantitation of WT and FAK^{KO} pericyte angiogenesis protein expression profiles, after co-culture with B16F0 cells. Bar chart represents mean densitometric reading of protein profiler WT (white bars) or FAK^{KO} (black bars)±s.e.m., n=4 WT and 6 FAK^{KO} experimental repeats. *p=0.016 (MCP-1), *p=0.0136 (NOV), ***p=0.0005 (Cyr61), *p=0.0884 (pentraxin-3). Two-sided Students *t* test. Red box, Cyr61 levels. (**b**) Gas6 ELISA. Depletion of Cyr61 in FAK^{KO} pericytes (FAK-null;Cyr61^{KO}) does not affect secretion of Gas6 compared with control FAK^{KO} pericytes (FAK-null;Cas9). Bar chart shows mean±sd, n=2 experimental repeats. Source data are provided as a Source data file.

Supplementary Figure 10 Lechertier *et al.*

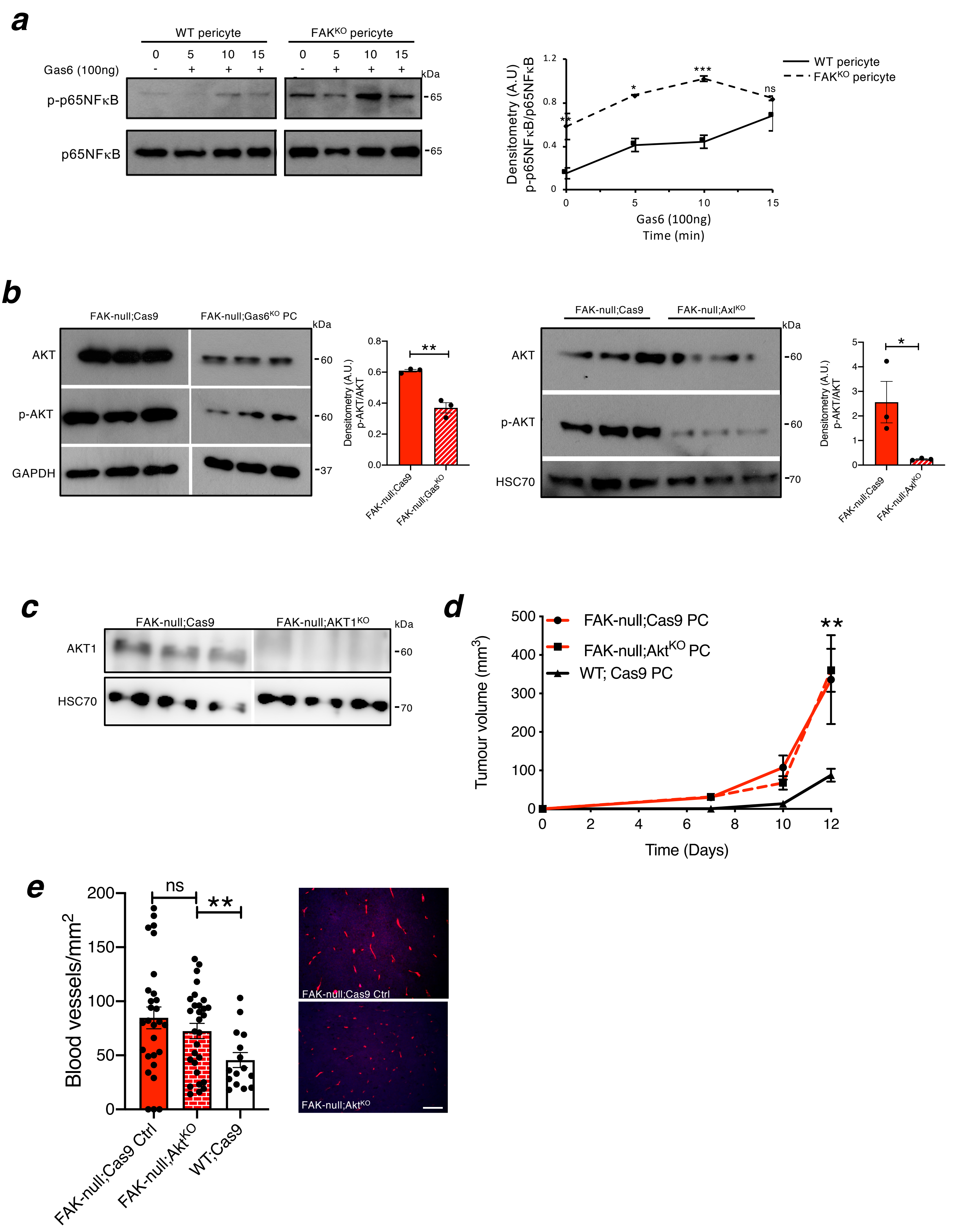

Supplementary Figure 10: **Increased phosphorylation of AKT in FAK^{KO} pericytes does not affect tumour growth. (a)** WT and FAK^{KO} pericytes were stimulated with Gas6 (100ng/ml for 5, 10 and 15 min) and lysates analysed by Western blot for p-p65NFκb and total p65NFκb. Graph represent the densitometric quantitation of p-p65NFκB/p65NFκb ratio±s.e.m. n=3 experimental repeats, *p=0.01, **p=0.02, ***p=0.0039. Two-sided Students *t* test. **(b)** Gas6 and Axl depletion in FAK^{KO} pericytes reduces p- and total-AKT levels. Bar charts represent densitometric quantitation of p-AKT/AKT±s.e.m.; n= 3 experimental repeats, **p=0.0022, *p=0.051. Two-sided Students *t* test. **(c)** AKT depletion in FAK^{KO} pericytes was confirmed by western blot. N=3 experimental repeats. **(d, e)** Tumour growth is not decreased after *AKT* depletion in FAK^{KO} pericytes after co-injection with B16F0 tumour cells. Line graph shows mean tumour volume (mm^3)±s.e.m.; **p=0.0045. One way ANOVA. Bar chart represent mean no. blood vessels (mm^2)±s.e.m.; **p=0.0034, ns= not significant. Two-sided Students *t* test. FAK-null;Cas9: n= 9 mice, FAK-null;AKT^{KO}; n= 7 mice, WT; n= 9 mice. Representative images show endomucin staining of tumour blood vessels. Scale bar, 100 μm. Source data are provided as a Source data file.

Supplementary Figure 11

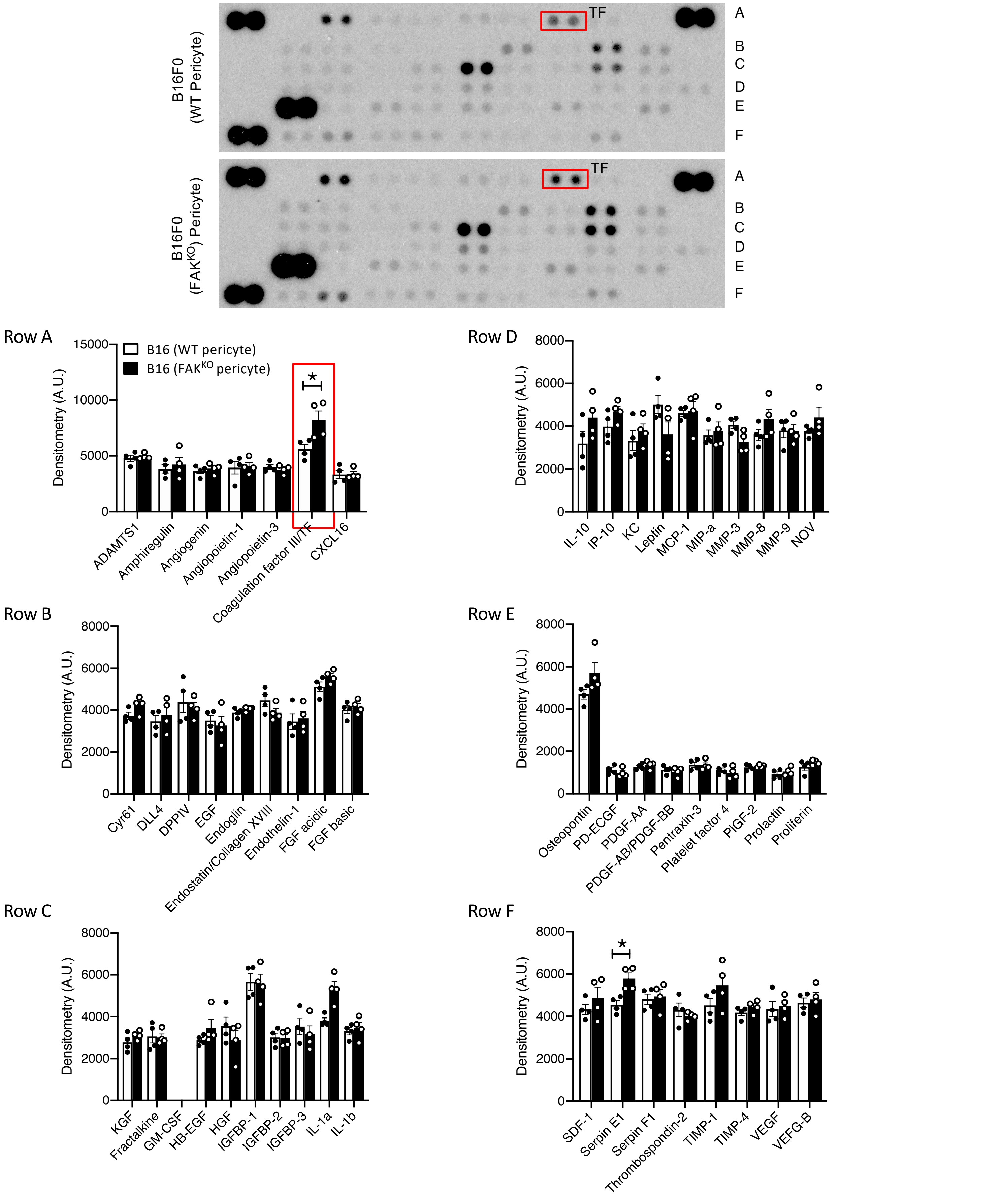

Supplementary Figure 11: **Angiogenesis protein expression dot blot array results from B16F0 after co-culture with WT or FAK^{KO} pericytes.** Representative angiogenesis arrays and quantitation of B16F0 proteins after co-culture with WT and FAK^{KO} pericytes. Bar chart represents mean densitometric reading of protein profiler B16 (WT pericytes) (white bars) or B16 (FAK^{KO}pericytes) (black bars)±s.e.m., n=4 experimental repeats. *p=0.0295 (TF), *p=0.01 (Serpin E1). Two-sided Students *t* test. Red box, TF levels. Source data are provided as a Source data file.

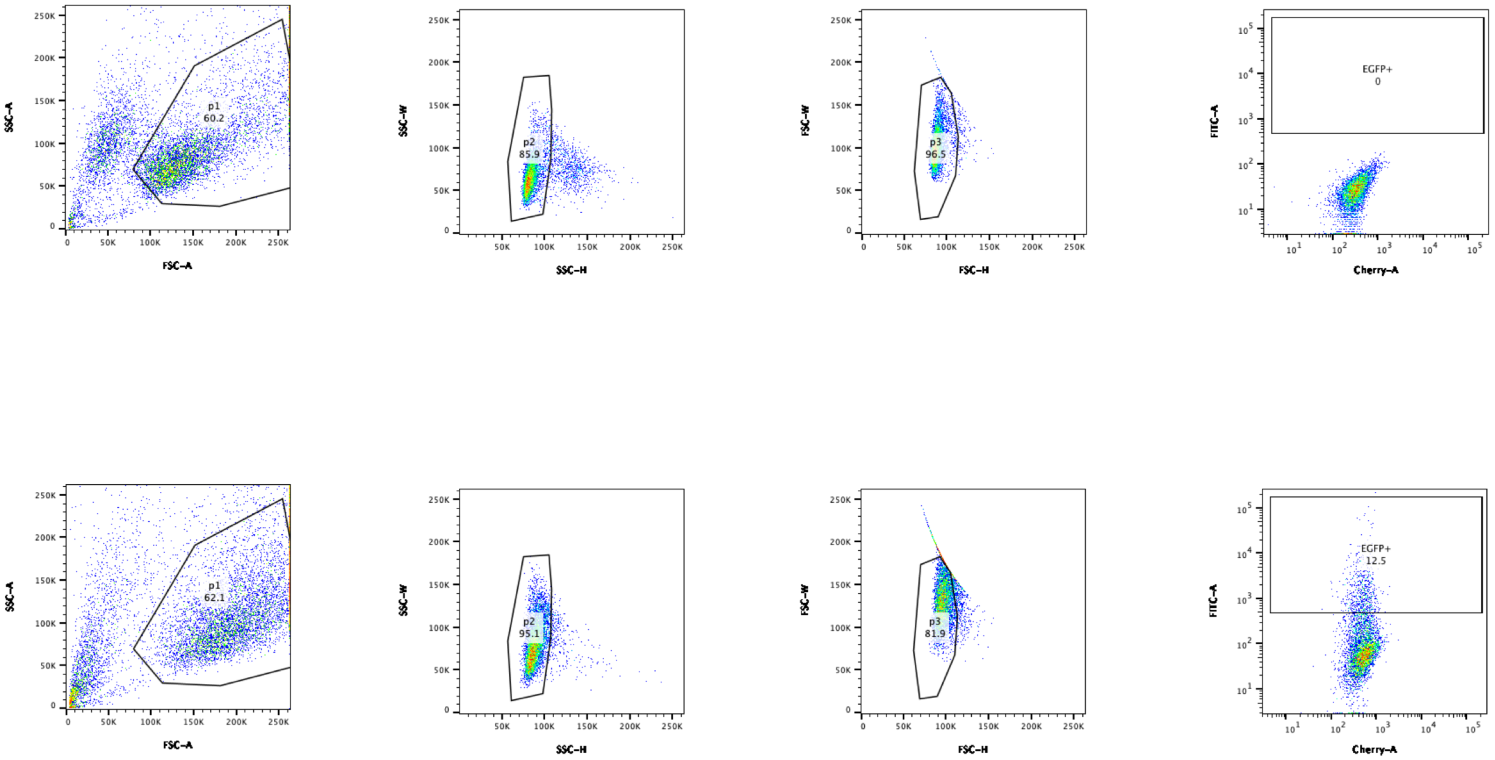

***Supplementary Figure 12:* Gating strategy for primary pericytes and B16F0 after CRISPR/Cas9 transfection**. Representative gating for viable pericytes or B16F0 cells via forward-scatter area (FSC-A) and side-scatter area (SSC-A), resulting in population P1. P1 population cells were gated via SSC-W and SSC-H and FSC-W and FSC-H to exclude doublet cells, resulting in population P2 and P3. Transfected cell population was gated via EGFP (488-nm) filter sets inside gate P3. Cells were analysed by flow cytometry at 48 h after transfection.

Supplementary Figure 13

Uncropped scans for Figure 2

f

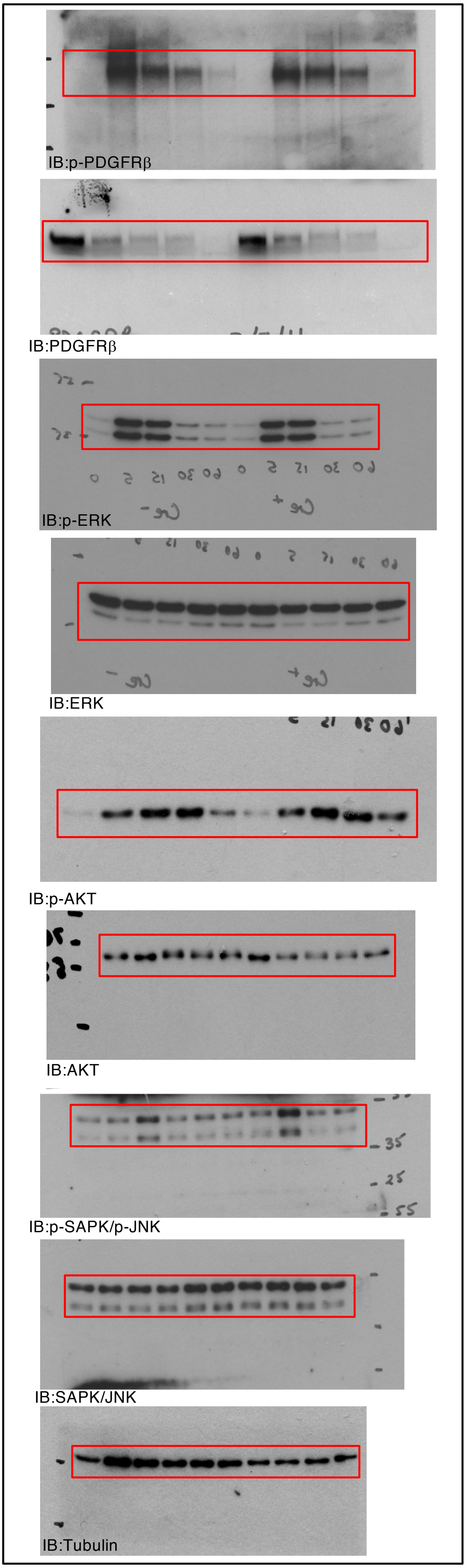

g

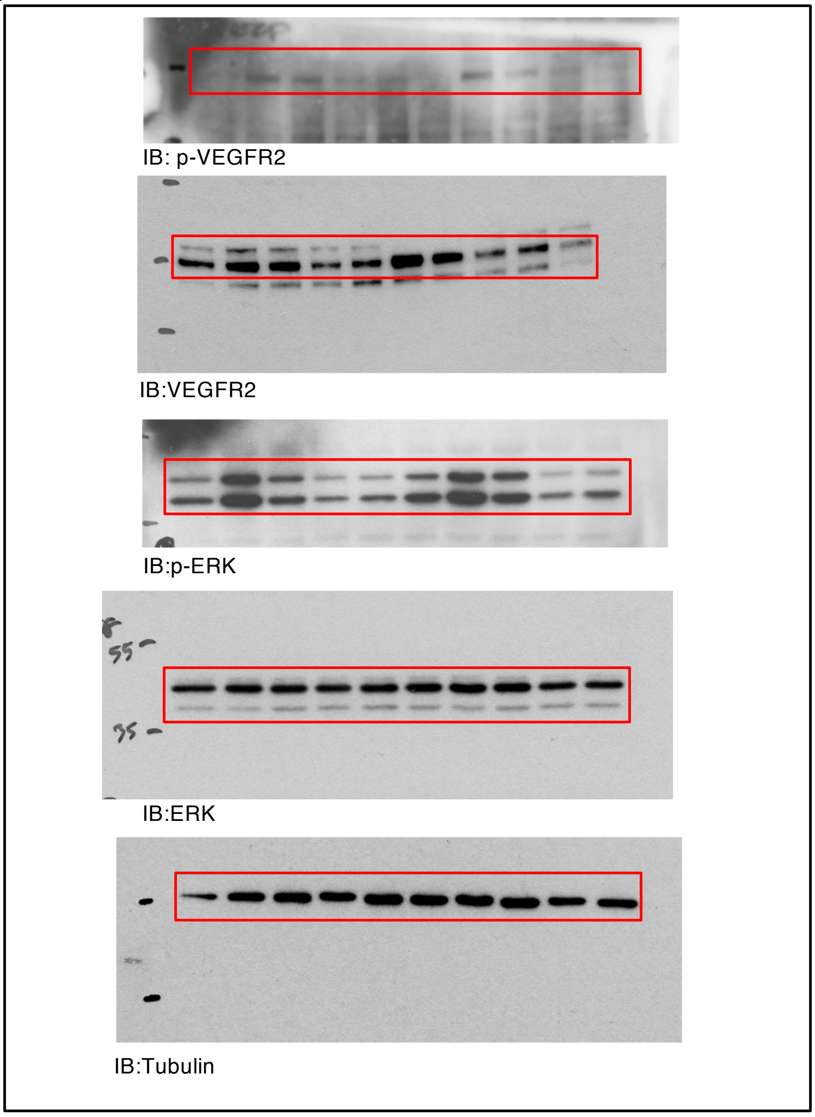

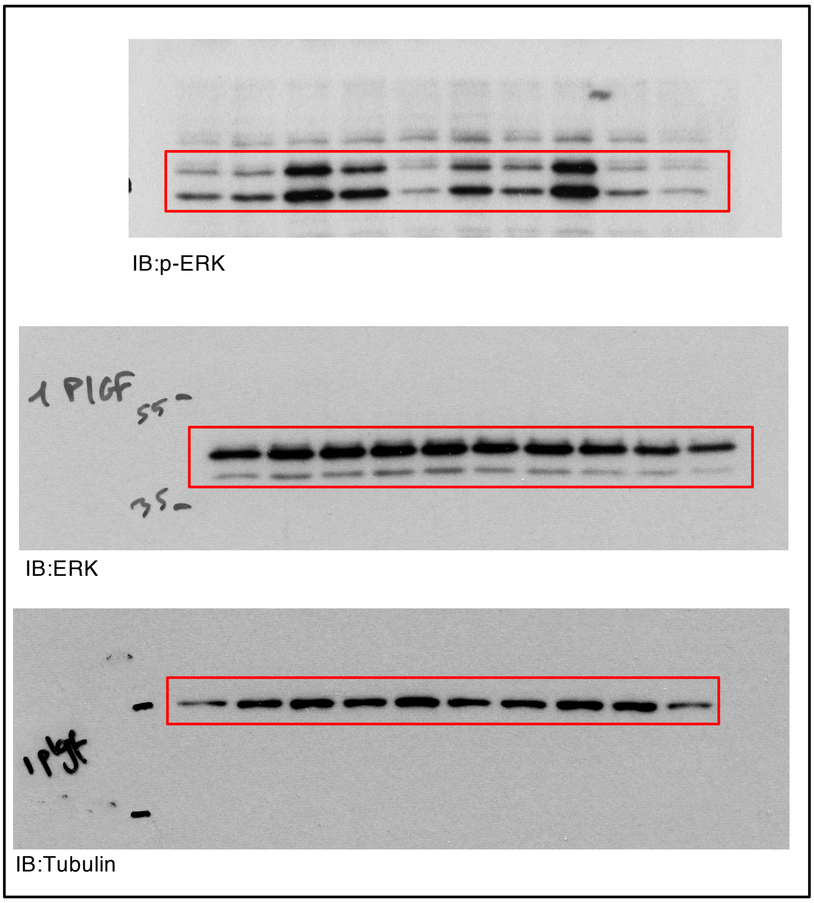

Uncropped scans for Figure 3

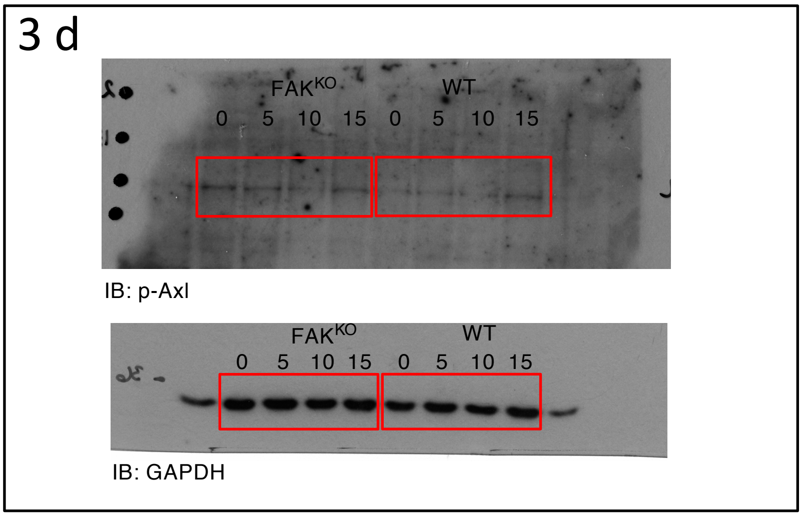

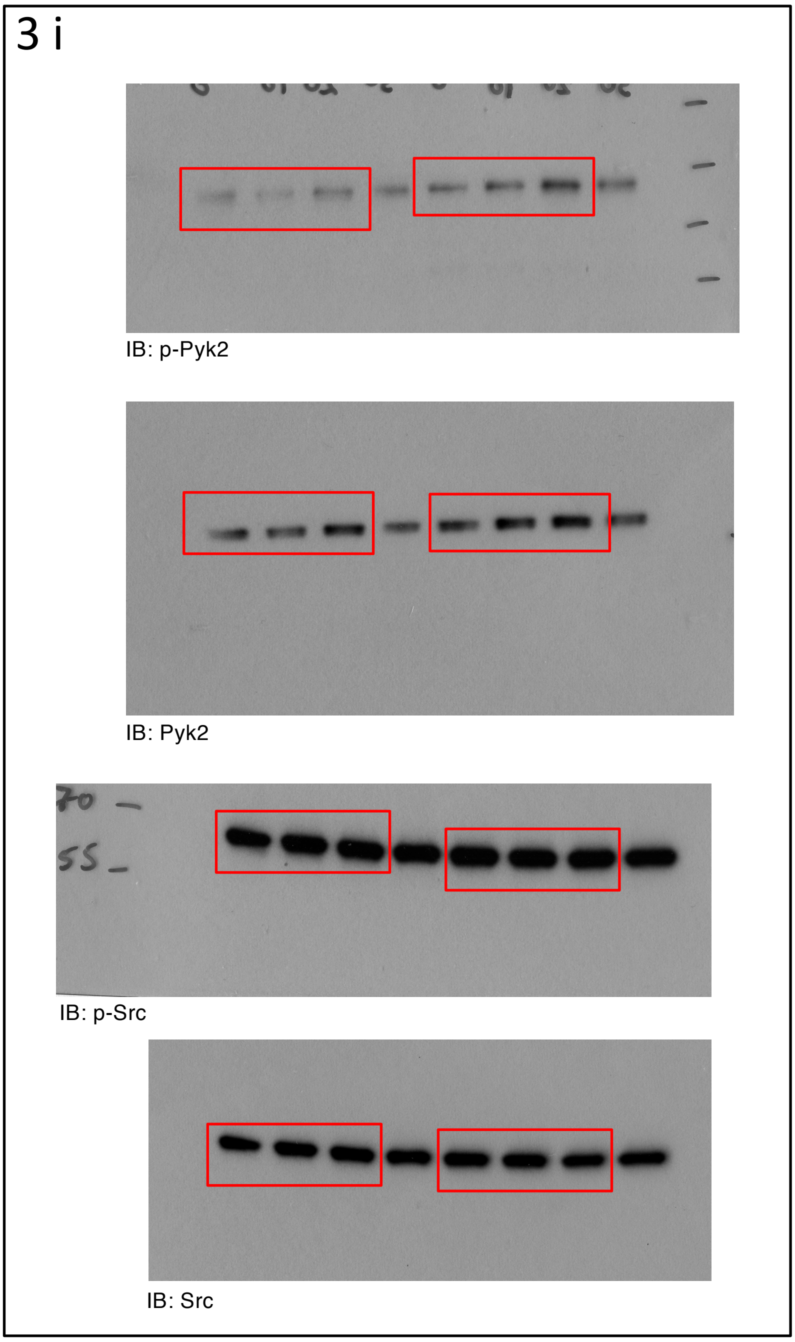

Uncropped scans for Figure 4

a

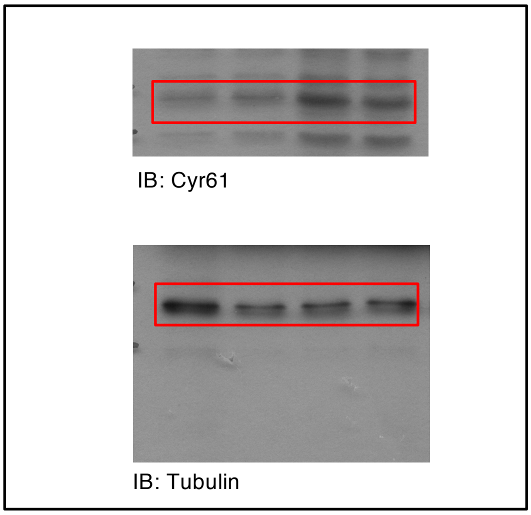

b

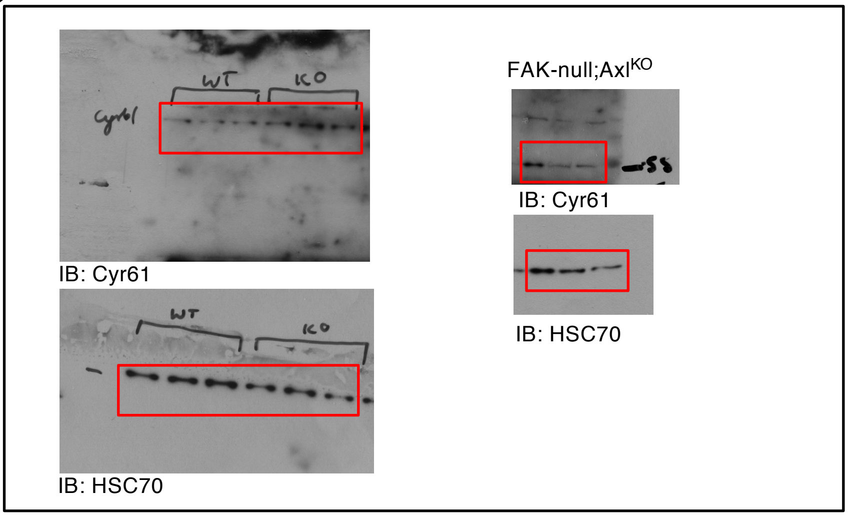

d

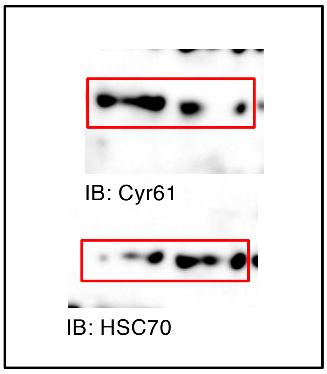

f

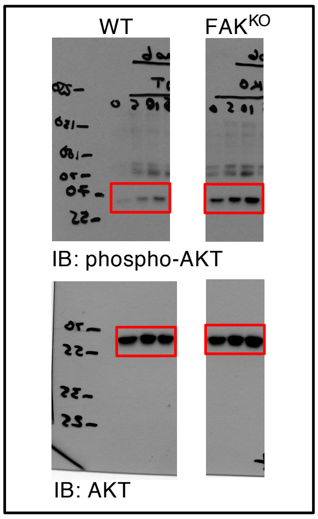

g

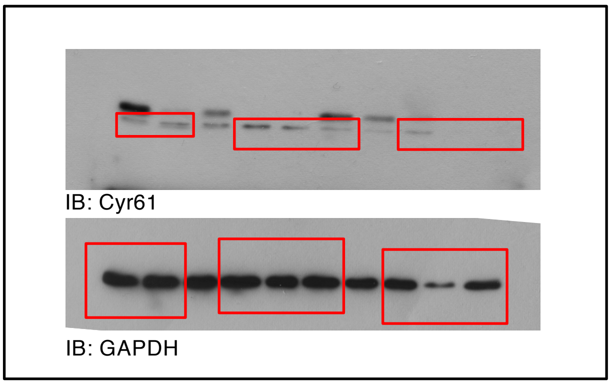

h

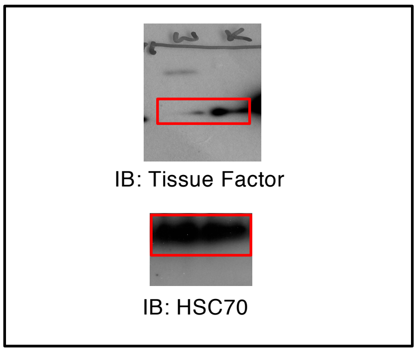

i

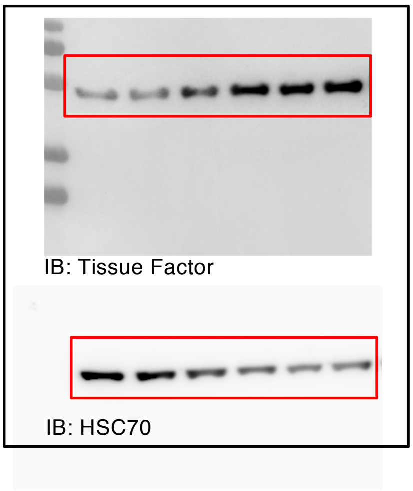

j

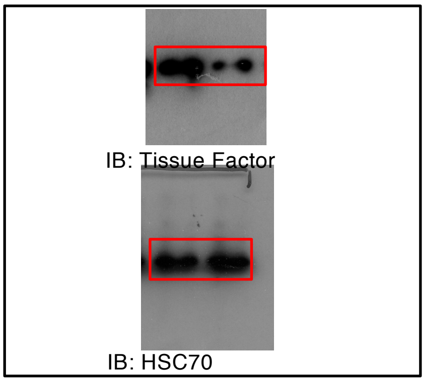

Uncropped scans for Supplementary Figure 1.e

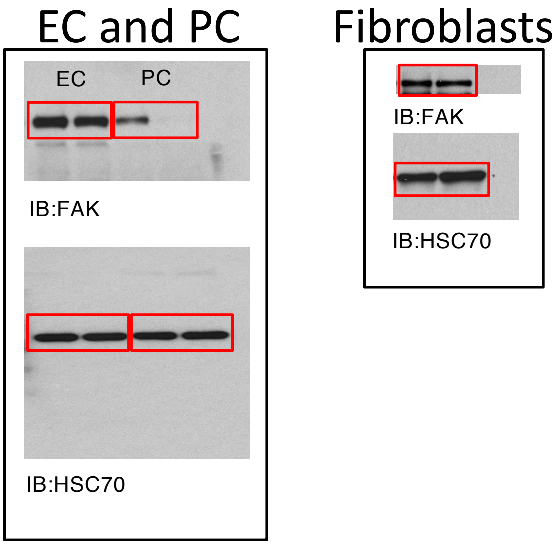

Uncropped scans for Supplementary Figure 7

a

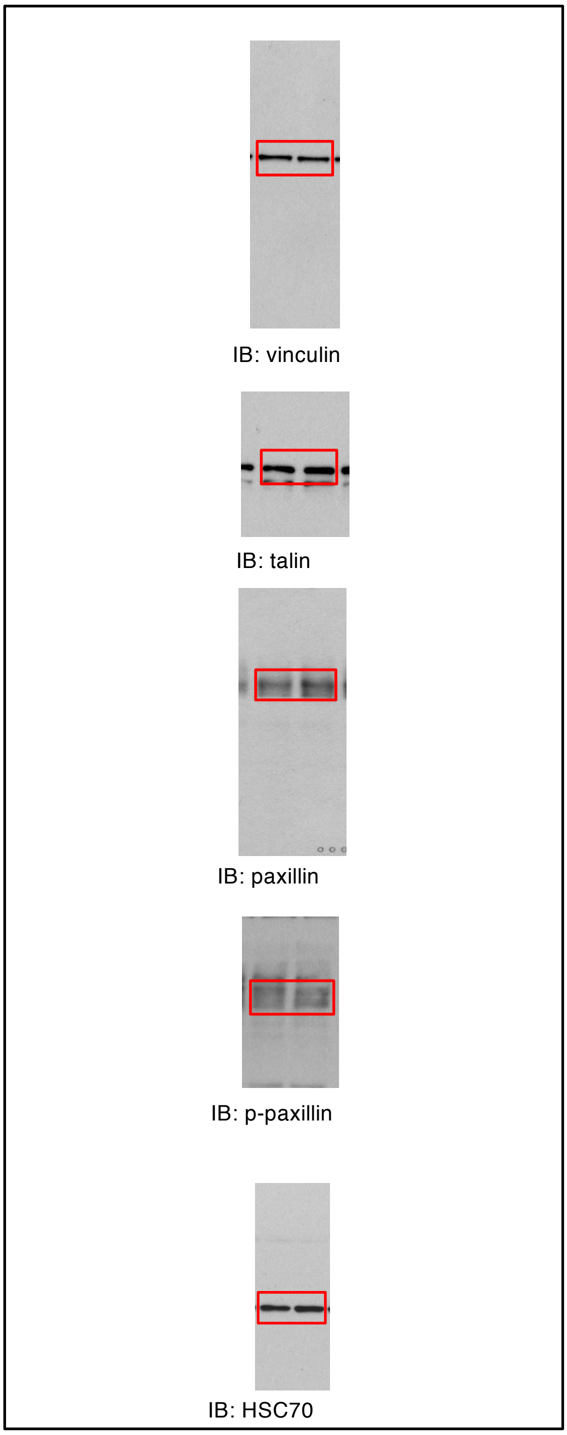

b

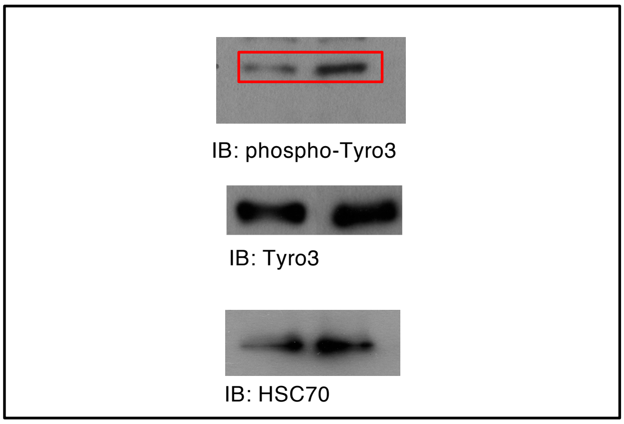

Uncropped scans for Supplementary Figure 8

8c

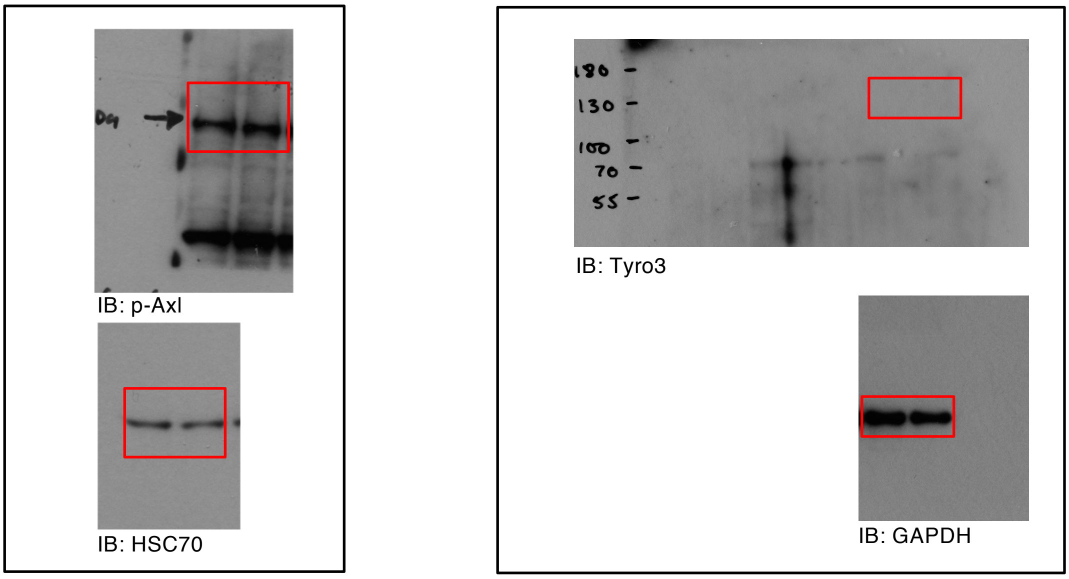

Uncropped scans for Supplementary Figure 10

a

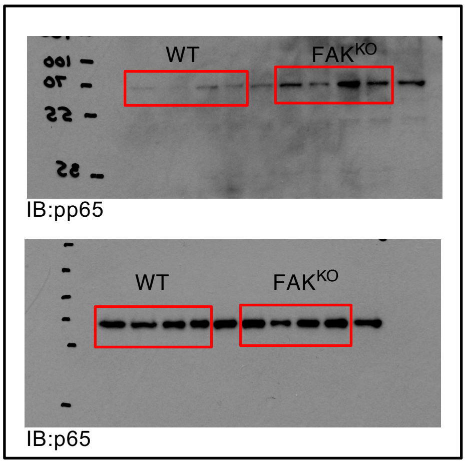

b – left panel

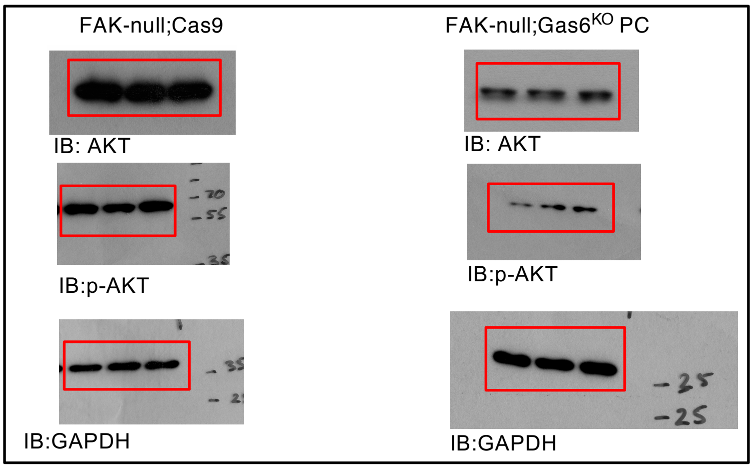

b – right panel

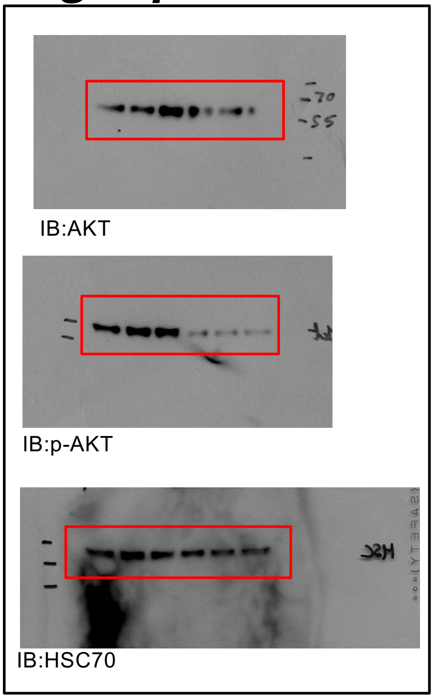

c

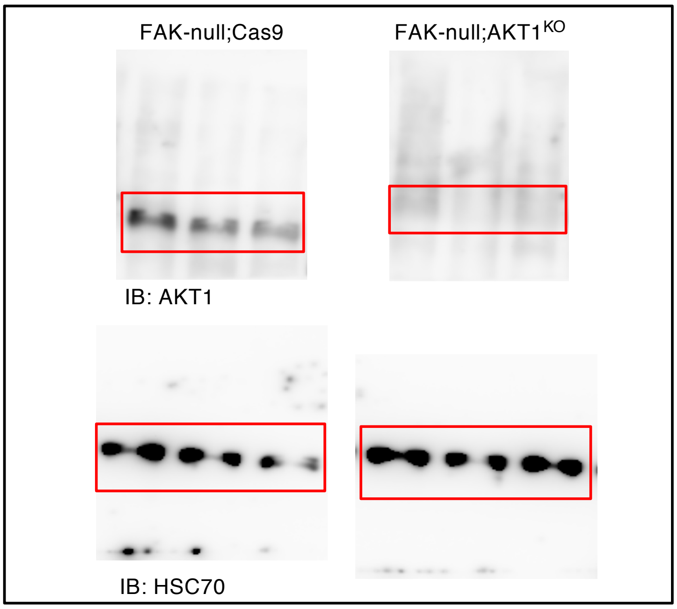

Supplementary Methods

Blood vessel perfusion

Tumour blood vessel perfusion was performed using an intravenous injection of Phycoerythrin labelled PECAM antibody (100 mg) (BioLegend) 10 minutes before tumour excision. The percentage of perfused tumour blood vessel was calculated by the ratio between the number of PE-PECAM positive and the total number of tumour blood vessels stained for endomucin (1:100).

Immunostaining

Five µm frozen sample sections were air-dried for 10 min, washed once in PBS, fixed in acetone for 10 minutes at -20 ° C, washed in PBS three times and then blocked with 5 % normal goat serum for 30 minutes at room temperature. After blocking, sections were incubated with primary antibodies overnight at 4 ° C. Primary antibodies used were directed against endomucin (Santa Cruz, V7C7, 1:100), NG2 (AB5320, Millipore, 1:100), Laminin (L9393, Sigma, 1:100), CD45 (10558, Abcam, 1:100) and F4/80 (MCA497GA, ABSerotec, 1:100). Sections were then washed with PBS and incubated with Alexa-Fluor®-conjugated secondary antibodies (Invitrogen) for 45 minutes at room temperature before being mounted using Prolong® Gold anti-fade reagent (Invitrogen). A Zeiss AxioPlan microscope and AxioVision software were used for imaging the slides.

For PDGFRB immunostaining of tumour sections, harvested tumours were fixed with 4% PFA in PBS at 4 degrees for 2-4 hours. The tissues were dehydrated overnight with 20 % sucrose solution in PBS at 4 degrees and embedded in tissue freezing media. The tissue sections were blocked and permeabilised with 5 % goat serum in PBST (0.3% Triton X-100 in PBS) for 1hr at RT. The tissue was incubated with primary antibody (1:200, APB5 clone for PDGFRβ) in blocking buffer at 4°C overnight. The following day, the tissue was washed 3 times with PBS then incubated with fluorescent conjugated secondary antibody (1:1000 dilution) for 1 hr at room temperature. Tissue was washed 6 times with PBS, once with water and mounted with Prolong Gold.

Blood vessel perimeter measurements

The perimeter of endomucin-positive blood vessels was quantified using the Axiovision™ software linear measuring tool.

Immune cell infiltration

Immunostaining of immune cells from B16F0 tumour sections was performed as described previously[49].
