# Supplementary material for: Pericyte FAK negatively regulates Gas6/Axl signalling to suppress tumour angiogenesis and tumour growth
Source: Nat Commun. 2020 Jun 4;11:2810. doi: 10.1038/s41467-020-16618-6 (PMC7272651; doi:10.1038/s41467-020-16618-6)
Supplement: Supplementary file 2 — Reporting Summary [file 41467_2020_16618_MOESM2_ESM.pdf]

nature research

Corresponding author(s): K.HODIVALA-DILKE

Last updated by author(s): 14/04/20

Reporting Summary

Nature Research wishes to improve the reproducibility of the work that we publish. This form provides structure for consistency and transparency in reporting. For further information on Nature Research policies, see Authors & Referees and the Editorial Policy Checklist.

Statistics

For all statistical analyses, confirm that the following items are present in the figure legend, table legend, main text, or Methods section.

n/a	Confirmed	
☐	☒	The exact sample size (n) for each experimental group/condition, given as a discrete number and unit of measurement
☐	☒	A statement on whether measurements were taken from distinct samples or whether the same sample was measured repeatedly
☐	☒	The statistical test(s) used AND whether they are one- or two-sided<br>*Only common tests should be described solely by name; describe more complex techniques in the Methods section.*
☒	☐	A description of all covariates tested
☒	☐	A description of any assumptions or corrections, such as tests of normality and adjustment for multiple comparisons
☐	☒	A full description of the statistical parameters including central tendency (e.g. means) or other basic estimates (e.g. regression coefficient) AND variation (e.g. standard deviation) or associated estimates of uncertainty (e.g. confidence intervals)
☐	☒	For null hypothesis testing, the test statistic (e.g. F, t, r) with confidence intervals, effect sizes, degrees of freedom and P value noted<br>*Give P values as exact values whenever suitable.*
☒	☐	For Bayesian analysis, information on the choice of priors and Markov chain Monte Carlo settings
☒	☐	For hierarchical and complex designs, identification of the appropriate level for tests and full reporting of outcomes
☒	☐	Estimates of effect sizes (e.g. Cohen's d, Pearson's r), indicating how they were calculated

Our web collection on statistics for biologists contains articles on many of the points above.

Software and code

Policy information about availability of computer code

Data collection	ImageJ software (V1.51) was used to quantify Western blot images, analyse HUVEC bead sprouting, Gas6 immunostaining, p-Axl immunostaining and single cell migration for the Dunn chamber assay<br>Images obtained from spinning disc were processed and analysed using Fiji (V1.51)<br>Axiovision Rel (V4.9.1) software was used to capture IF/IHC images on the Zeiss Axioplan microscope<br>qRT-PCR data was analysed using StepOne Real Time PCR machine and software<br>ELISA and adhesion assay data was analysed using the Tecan plate reader<br>Prism V8.3.0 software was used for statistical analysis of data<br>IncuCyte Zoom system and software (v2018B) was used for the proliferation assay<br>Micro Manager acquisition software (v2.0 gamma) was used to collect images for the Dunn chamber assay<br>Motion analysis of single cell migration was analysed using Wolfram Mathematica v7.0 software
Data analysis	sgRNAs were designed using the CRISPOR algorithm (http://crispor.tefor.net)

For manuscripts utilizing custom algorithms or software that are central to the research but not yet described in published literature, software must be made available to editors/reviewers. We strongly encourage code deposition in a community repository (e.g. GitHub). See the Nature Research guidelines for submitting code & software for further information.

Data

Policy information about availability of data

All manuscripts must include a data availability statement. This statement should provide the following information, where applicable:

- Accession codes, unique identifiers, or web links for publicly available datasets
- A list of figures that have associated raw data
- A description of any restrictions on data availability

A Data availability statement has been included at the end of the manuscript, before the refs.

"Data availability statement
All the relevant data that support the findings of this study are available from the corresponding author on request."

Field-specific reporting

Please select the one below that is the best fit for your research. If you are not sure, read the appropriate sections before making your selection.

☒ Life sciences ☐ Behavioural & social sciences ☐ Ecological, evolutionary & environmental sciences

For a reference copy of the document with all sections, see nature.com/documents/nr-reporting-summary-flat.pdf

Life sciences study design

All studies must disclose on these points even when the disclosure is negative.

Sample size	Samples sizes were determined according to power calculations. In consultation with our in-house statisticians, Prof Duffy and Dr North, we have done pilot studies to determine the numbers of animals required to provide statistical significance in our results. Prof Duffy has done the power calculations to estimate that we will require 10 mice/cohort. Using a two-sided test with a 5% significance level, his calculations predict 85-90% power to reject the null hypothesis of no difference assuming the two genotypes differ by the magnitudes that we have observed in similar experiments. For tumour growth and tumour response studies two-way ANOVA or Student's t-test will be used.
Data exclusions	Inclusion criteria: within each experiment animal groups were the same age, sex, genetic strain and maintained under the same conditions. Exclusion criteria: mice were excluded from the experiment only if unexpected adverse effects were observed.
Replication	All experimental data are given including replicates. Details of experimental replicates are given in the figure legends. All reported attempts at replication were successful.
Randomization	Within each experiment, mice were randomly assigned to different groups to avoid bias. In other experiments cells were randomly assigned to groups to avoid bias. All histological analyses were done in a blinded fashion.
Blinding	All data collection and analysis was blinded.

Reporting for specific materials, systems and methods

We require information from authors about some types of materials, experimental systems and methods used in many studies. Here, indicate whether each material, system or method listed is relevant to your study. If you are not sure if a list item applies to your research, read the appropriate section before selecting a response.

Materials & experimental systems

n/a	Involved in the study	
☐	☒	Antibodies
☐	☒	Eukaryotic cell lines
☒	☐	Palaeontology
☐	☒	Animals and other organisms
☐	☒	Human research participants
☒	☐	Clinical data

Methods

n/a	Involved in the study	
☒	☐	ChIP-seq
☐	☒	Flow cytometry
☒	☐	MRI-based neuroimaging

Antibodies

Antibodies used

Endomucin (clone V.7C7, Santa Cruz, cat no. sc-65495)
alpha-smooth muscle actin Cy3-conjugated (clone 1A4, Sigma-Aldrich, cat no. C6198)
alpha-smooth muscle actin (Clone 1A4, mouse monoclonal, Sigma-Aldrich, cat no. A2547)
AlexaFluor 488 goat anti-rabbit IgG (ThermoFisher Scientific, cat no. A-11008)
PE-PECAM (MEC13.3, Biolegend, cat no. 102507)
HSC70 (Clone B6, mouse monoclonal, Santa Cruz, cat no. sc7298)
GAPDH (Clone 6C5, mouse monoclonal, Millipore, cat no. MAB374)
FAK (Cell Signaling, cat no.3285)
PDGFR-b for immunostaining (Clone APB5, rat monoclonal, ThermoFisher, cat no. 14-1402-82)
PDGFR-b for FACs (Clone APB5, rat monoclonal, Novus Biologicals, cat no. NBP1-43349)
PDGFR-b (Clone 28E1, rabbit monoclonal, Cell Signaling cat no. 3169)
Phospho-PDGFR-b (Cell Signaling cat no. 3161)
ERK (Cell Signaling cat no. 9102)
Phospho-ERK (Cell Signaling cat no. 9101)
SAPK/JNK (Cell Signaling, cat no. 9252)
Phospho-SAPK/JNK (Cell Signaling, cat no. 9251)
Tubulin (Cell Signaling, cat no. 2146)

Hoechst (Sigma, cat no.14533)
VEGFR2 (Clone D5B1, rabbit monoclonal, Cell Signaling, cat no. 9698)
Phospho-VEGFR2 (Clone 19A10, rabbit monoclonal, Cell Signaling, cat no. 2478)
Phospho-Axl for immunostaining (R&D, cat no. AF2228)
Phospho-Axl for WB (E-AB-34244, Elabscience)
Axl (R&D, cat no. AF854)
Pyk2 (Clone H364, rabbit monoclonal, Cell Signaling, cat no. 3090)
Phospho-Pyk2 (Cell Signaling, cat no. 3291)
AKT (Cell SIgnaling, cat no. 9611)
Phospho-AKT (Clone 11E7, rabbit monoclonal, Cell SIgnaling, cat no. 4685)
SV40 (Clone Pab101, mouse monoclonal, Santa Cruz, cat no. sc-147)
Ki67 (Abcam, cat no. ab15580)
Src (Cell Signaling, cat no. 2108)
Phospho-Src (Clone D49G4, rabbit monoclonal, Cell Signaling, cat no. 6943)
Cyr61 (Millipore, cat no. ABC102)
Tissue Factor (Clone 355220, rat monoclonal, R&D, cat no. MAB3178)
Gas6 (LS Bio, cat no. LS-B13094)
BS-1 lectin (Sigma, cat no. L9381)
Alexa Fluor goat anti0rabbit 488 (
NG2 (Millipore, cat no. AB5320)
Laminin (Sigma, cat no. L9393)
VE-Cadherin (Clone BV14, rat monoclonal, cat no. NBP1-43347)
CD45 (Abcam, cat no. 10558)
F480 (Clone A3-1 rat, monoclonal, ABSerotec, cat no. MCA497GA)
Vinculin (Clone E1E9V rabbit monoclonal, Cell Signaling, cat no. 13901)
Talin (Abcam, cat no. 71333)
Paxillin (Clone 5H11, mouse monoclonal, Life Technologies, cat no. AHO0492)
Phospho-paxillin (Life Technologies, cat no. 44-722G)
Tyro (Clone D38C6, rabbit monoclonal, Cell Signaling, cat no. 5585)
Phospho-Tyro3 (ThermoFisher Scientific, cat no. PA5-40270)
P65NFKB (Clone D14E12, rabbit monoclonal, Cell Signaling, cat no. 8242)
Phospho-p65NFKB (Clone 93H1, rabbit monoclonal, Cell Signaling, cat no. 3033)
AKT1 (Clone 2H10, mouse monoclonal, Cell Signaling, 2967)
Rhodamine phalloidin (ThermoFisher Scientific, cat no. R415)
FCgRII/III (Clone Ab93, Millipore, cat no. MABF838)
ICAM-2 (Pharmingen, cat no. 553326, clone3C4)
Anti-rabbit biotin (DAKO, cat no. E0353)
Alexa fluor goat anti-rabbit 488 (Invitrogen, cat no. A32731)

Validation	All antibodies were validated by the commercial supplier. All validation statements are found on the respective antibody website.

Eukaryotic cell lines

Policy information about cell lines

Cell line source(s)	B16F0, ATTC cat no. CRL- 6322<br>LLC, ATTC, cat no. CRL-1642
Authentication	The lines used were all derived from mouse. We have not authenticated these lines ourselves. See suppliers websites for authentication details.
Mycoplasma contamination	All cell lines used were mycoplasma free
Commonly misidentified lines (See ICLAC register)	None as far as we know

Animals and other organisms

Policy information about studies involving animals; ARRIVE guidelines recommended for reporting animal research

Laboratory animals	For animals bred in-house- health screens (quarterly) were conducted in accordance with FELASA guidelines for health monitoring of rodent colonies, to confirm their free statues of known pathogens in accordance with FELASA screens. No clinical signs were detected. Animals were housed in groups of 4-6 mice per individually ventilated cage in a 12 h light dark cycle (06:30-18:30 light; 18:30-06:30 dark), with controlled room temperature (21 ± 1 °C) and relative humidity (40-60 %). The cages contained 1-1.5 cm layer of animal bedding, and with environmental enrichment including cardboard Box-tunnel and crinkled paper nesting material. Animals had access to food and water ad libitum.<br>Species: Mouse.<br>Strain:C57/Bl6J/129<br>Sex: Male and female<br>Age: 8-10 weeks

For animals bred in-house- health screens (quarterly) were conducted in accordance with FELASA guidelines for health monitoring of rodent colonies, to confirm their free statues of known pathogens in accordance with FELASA screens. No clinical signs were detected. Animals were housed in groups of 4-6 mice per individually ventilated cage in a 12 h light dark cycle (06:30-18:30 light; 18:30-06:30 dark), with controlled room temperature (21 ± 1 °C) and relative humidity (40-60 %). The cages contained 1-1.5 cm layer of animal bedding, and with environmental enrichment including cardboard Box-tunnel and crinkled paper nesting material. Animals had access to food and water ad libitum.
Species: Mouse.
Strain:C57/Bl6J/129
Sex: Male and female
Age: 8-10 weeks

Wild animals	This study did not involve wild animals
Field-collected samples	This study did not involve samples collected from the field
Ethics oversight	All procedures were approved by our local animal ethics committee, Queen Mary University of London, and were executed in accordance with United Kingdom Home Office regulations. All animal work was carried out in accordance with ARRIVE Guidelines.

Note that full information on the approval of the study protocol must also be provided in the manuscript.

Human research participants

Policy information about studies involving human research participants

Population characteristics	N/A
Recruitment	N/A
Ethics oversight	Melanoma samples were covered by East London and City Health Authority Ethical Approval (07/Q0604/23 MM: East London and City Health Authority: 'Molecular mechanisms in the pathogenesis of malignant melanoma', with a minor amendment in March 2018: ReDa 005044Q1.

Note that full information on the approval of the study protocol must also be provided in the manuscript.

Flow Cytometry

Plots

Confirm that:

☒ The axis labels state the marker and fluorochrome used (e.g. CD4-FITC).

☒ The axis scales are clearly visible. Include numbers along axes only for bottom left plot of group (a 'group' is an analysis of identical markers).

☒ All plots are contour plots with outliers or pseudocolor plots.

☒ A numerical value for number of cells or percentage (with statistics) is provided.

Methodology

Sample preparation	Pericytes and B16F0 tumour cells were grown in culture to the relevant confluency, then trypsinised prior to transfection with CrisprCas lentivirus.
Instrument	FACS Aria III flow cytometer (BD Biosciences)
Software	Data were transferred and analysed using the FlowJo software (Tree Star, Oregon, USA) version v10.0.8.
Cell population abundance	No cell sorting experiments were performed and thus cell population abundance is not relevant to our study
Gating strategy	Viable pericytes and B16F0 cells were gated via forward scatter area (FSC-A) and side scatter area (SSC-A) resulting in population P1. P1 population cells were gated via SSC-W and SSC-H and FSC-W and FSC-H to exclude doublet cells, resulting in population P2 and P3. Tansfected cell populations were gated via EGFP (488-nm) filter sets inside gate P3.

☒ Tick this box to confirm that a figure exemplifying the gating strategy is provided in the Supplementary Information.
